# Distinct sensorimotor mechanisms underlie the control of grasp and manipulation forces for dexterous manipulation

**DOI:** 10.1038/s41598-023-38870-8

**Published:** 2023-07-25

**Authors:** Yen-Hsun Wu, Marco Santello

**Affiliations:** School of Biological and Health Systems Engineering, Arizona State University, Tempe, AZ 85287 USA

**Keywords:** Motor control, Sensorimotor processing

## Abstract

Dexterous manipulation relies on the ability to simultaneously attain two goals: controlling object position and orientation (pose) and preventing object slip. Although object manipulation has been extensively studied, most previous work has focused only on the control of digit forces for slip prevention. Therefore, it remains underexplored how humans coordinate digit forces to prevent object slip and control object pose simultaneously. We developed a dexterous manipulation task requiring subjects to grasp and lift a sensorized object using different grasp configurations while preventing it from tilting. We decomposed digit forces into manipulation and grasp forces for pose control and slip prevention, respectively. By separating biomechanically-obligatory from non-obligatory effects of grasp configuration, we found that subjects prioritized grasp stability over efficiency in grasp force control. Furthermore, grasp force was controlled in an anticipatory fashion at object lift onset, whereas manipulation force was modulated following acquisition of somatosensory and visual feedback of object’s dynamics throughout object lift. Mathematical modeling of feasible manipulation forces further confirmed that subjects could not accurately anticipate the required manipulation force prior to acquisition of sensory feedback. Our experimental approach and findings open new research avenues for investigating neural mechanisms underlying dexterous manipulation and biomedical applications.

## Introduction

Imagine grasping a glass of water and lifting it for a sip. To successfully perform this task, the central nervous system (CNS) must solve a complex problem: you do not want the glass to slip, and therefore may want to squeeze it hard. However, you also want to accurately tilt the glass so that you can drink from it without spilling its content, which requires dynamically changing the digit force distribution. Importantly, digit forces must be coordinated to simultaneously attain these task goals. Although humans have an exquisite ability to perform dexterous manipulation, how digit forces are coordinated to simultaneously prevent object slip and control object position and orientation (pose) has not been investigated. This gap in our knowledge is mainly due to the fact that, over the past four decades, most grasping studies have used tasks requiring subjects to modulate digit forces for preventing object slip as a function of object properties (e.g., mass^1,2^, and contact surface^3^), and/or environmental variability^4,5^, but were devoid of dexterity requirements associated with object pose control.

Andrew Gordon and colleagues first introduced a dexterity component by requiring subjects to coordinate digit forces to minimize the tilt of an inverted T-shaped object with an asymmetrical mass distribution^6^. For the grip device used by these authors, contact surfaces were aligned with the direction of gravity, while thumb and index fingertip faced each other in the same horizontal plane such that their force vectors were both horizontal and collinear. Here, the two orthogonal digit force components—normal and tangential to the contact surface (grip and load force, respectively)—are also perpendicular to and aligned with, respectively, the direction of gravity. If the contact surfaces remain aligned with the direction of gravity, grip force is entirely devoted to preventing object slip, whereas load force is devoted to object pose control. However, as the object tilts, a portion of the grip force will now contribute to object pose control, thereby preventing the separation of the functional role (object slip versus pose control) of digit forces. This example shows that traditional analyses developed to quantify digit force coordination to prevent object slip^7–16^ are not suitable to quantify digit force coordination when the manipulation task also has a dexterity component. We also note that when thumb and index finger force vectors are not horizontal and collinear, e.g., the thumb is higher or lower than the index fingertip, the above issue is amplified. This is because a portion of grip force is now devoted to generating a torque, thereby further contributing to object pose control^17^. The present work was designed to address these gaps and investigate humans’ ability to plan and execute digit forces for object slip prevention and pose control. Specifically, we used an approach that combines (a) a novel application of digit force analysis tools designed for robotic manipulation and (b) a dexterous task that requires simultaneous object slip prevention and tilt minimization at different grasp configurations.

In robotics, the set of contact forces required to manipulate an object are determined based on the desired manipulation goal and contact distribution^18,19^. This process relies on mathematically decomposing digit forces into two functional components^20^: manipulation force (*F*_*M*_) and grasp force (*F*_*G*_). *F*_*M*_ is the force responsible for object pose control which, in our task, corresponds to the object tilt minimization requirement. In contrast, *F*_*G*_—equivalent to the internal force—has no effect on object movement and its role is to prevent object slip. *F*_*G*_ is limited only by digit strength, object fragility, and contact surface condition. Therefore, this grasp-manipulation force decomposition algorithm can effectively disentangle the multiple roles played by digit forces when the task requires to simultaneously prevent object slip and control object pose.

**Figure 1:**
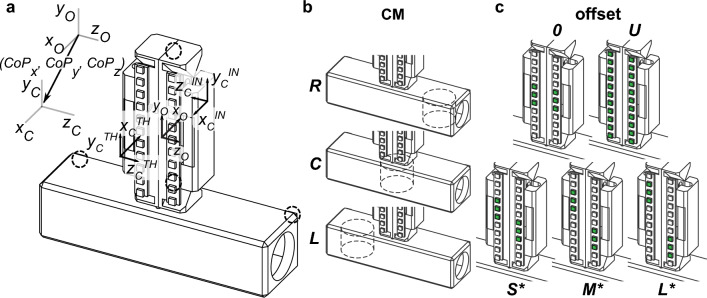
Grip device and experimental conditions. (**a**) We used a custom-made sensorized vertical handle mounted on a horizontal structure. The coordinate frames of the object (*x*_O_, *y*_O_, *z*_O_) and contact points (*x*_c_, *y*_c_, *z*_c_) at the thumb (TH) and index finger (IN) sides are shown. A column of LEDs was placed in front of each graspable surface. Object kinematics was tracked using four infrared LED markers (black dashed circles). (**b**) and (**c**) Experimental conditions consisted of (**b**) three centers of mass (CM; L, C and R denote left, center and right, respectively, relative to the subject) and (**c**) five vertical distances (offset) between the thumb and index fingertip. Offsets ‘0’ and ‘U’ denote zero thumb-index finger vertical distance and ‘unconstrained’ grasping, respectively. Small, medium, and large vertical distances are denoted by S, M and L, respectively. Examples of these three offsets (*) are shown for the Left CM condition whereby the thumb has to be positioned higher than the index finger (opposite offsets, i.e., index finger higher than the thumb, were used for the Right CM condition).

Regarding our dexterous manipulation task, we asked participants to grasp an instrumented inverted-T shape object (Fig. 1a) with thumb and index fingertip, lift it while preventing it from tilting, and hold it. We systematically changed the object’s mass distribution (CM; Fig. 1b) and the vertical distance between the digits (offset; Fig. 1c), i.e., grasp configurations. The former factor was introduced to add a dexterity component to our manipulation task as the asymmetrical object mass distribution requires subjects to generate a compensatory torque to lift the object straight. The rationale for increasing digit offset was that it changes the moment arm of normal digit forces, and therefore their contribution to object pose control. We analyzed digit forces at two task epochs, object lift onset and hold, as these two epochs engage distinct sensorimotor mechanisms^6,17,12^. Specifically, digit forces at object lift onset reflect feedforward control mechanisms, hence force planning, based on sensorimotor memory built through previous manipulations^17,21^. In contrast, digit forces used from object lift onset, throughout object lift and during object hold can be modulated through somatosensory and visual feedback. Therefore, decomposing digit forces into *F*_*M*_ and *F*_*G*_ allowed us to address how these two types of forces are planned and executed to simultaneously minimize the risk of object slip and tilt. We note that the digit force distribution used during static object hold, unlike the distribution used at object lift onset, benefits from the acquisition of feedback about the object dynamics acquired during the lift. As such, this static digit force distribution can be considered as the solution chosen by subjects to simultaneously prevent object slip and minimize object tilt based on the maximum amount of sensory feedback available. Therefore, the comparison between the digit force distributions during object hold versus lift onset allows to determine the extent to which the steady-state solution (object hold) can be anticipated before acquiring somatosensory feedback (object lift onset).

We expected subjects to plan a default *F*_*G*_ in a feedforward manner regardless of object CM and grasp configuration. This expectation is based on previous research showing that grip force required to prevent object slip is modulated to object properties before object lift onset after only a few manipulations^1,2^. Therefore, we hypothesized that *F*_*G*_ selected at object lift onset would approach *F*_*G*_ exerted throughout object lift and during static object hold. Unlike *F*_*G*_, the extent to which *F*_*M*_ can be planned in a feedforward manner is unknown as there are no studies that have decomposed *F*_*M*_ from *F*_*G*_ in dexterous manipulation tasks. Nevertheless, we envisioned two alternative outcomes. The first outcome consists of feedforward planning of *F*_*M*_ as described for *F*_*G*_ such that *F*_*M*_ exerted at object lift onset would approach *F*_*M*_ exerted during object hold. The alternative outcome considers the possibility that, at object lift onset, subjects would not be able to accurately predict the consequences of *F*_*M*_ on object pose throughout the lift. We hypothesized that subjects would be able to plan *F*_*M*_ in a feedforward fashion as expected for *F*_*G*_. The rationale for this hypothesis is that subject would be able to build a sensorimotor memory of previous hand-object dynamics and use it to control object pose through anticipatory control of *F*_*M*_ at object lift onset.

## Results

As our analyses involved a large number of variables, Table 1 shows all the variables and their definitions.

**Table 1:** List of variables and definitions.

Subscripts and Superscripts	
O	Coordinate frames fixed to the object
c	Coordinate frames at contact points
x, y, z	Individual components in the corresponding coordinate system
TH	Thumb
IN	Index finger
Variables	
*T*_*COM*_	Compensatory torque
*T*_*EXT*_	External torque caused by added mass
*T*_*RES*_	Resultant torque defined as the difference between *T*_*EXT*_ and *T*_*COM*_
*F*_*M*_	Manipulation force
^*z*^*F*_*M*_	Normal component of *F*_*M*_
^*y*^*F*_*M*_	Tangential component of *F*_*M*_
∆^*y*^*F*_*M*_	the difference between thumb and index finger ^*y*^*F*_*M*_
∆^*z*^*F*_*M*_	The difference between thumb and index finger ^*z*^*F*_*M*_
*F*_*G*_	Grasp force
*F*_*G*_^*min*^	Minimum *F*_*G*_ required to prevent object slip
^*z*^*F*_*G*_^*min*^	Normal component of *F*_*G*_^*min*^
*SM*_*G*_	Relative grasp safety margin
*F*_*G*_^*EX*^	Excessive grasp force defined as *F*_*G*_ above *F*_*G*_^*min*^
^*z*^*F*_*G*_^*EX*^	Normal component of *F*_*G*_^*EX*^
^*y*^*F*_*G*_^*EX*^	Tangential component of *F*_*G*_^*EX*^
^*z*^*F*_*G*_^*EX TH*^	Thumb ^*z*^*F*_*G*_^*EX*^
^*y*^*F*_*G*_^*EX TH*^	Thumb ^*y*^*F*_*G*_^*EX*^
*CV*|*F*_*M*_|	Across-trial coefficient of variation for *F*_*M*_
*CV*|*F*_*G*_^*EX*^|	Across-trial coefficient of variation for excessive *F*_*G*_

### Fast learning of dexterous manipulation task regardless of grasp configuration

We asked subjects to perform a dexterous manipulation of a sensorized object using a precision grip at different grasp configurations and object’s mass distributions (Fig. 1). Our task required coordination of grasp and manipulation forces to simultaneously prevent object slip and tilt, respectively (see Sect. "Materials and methods"). To determine the effects of digit offset, linear mixed-effects models (LMMs) built with random intercepts for the random-effect factor *Subject* were fitted for all dependent variables.

**Figure 2:**
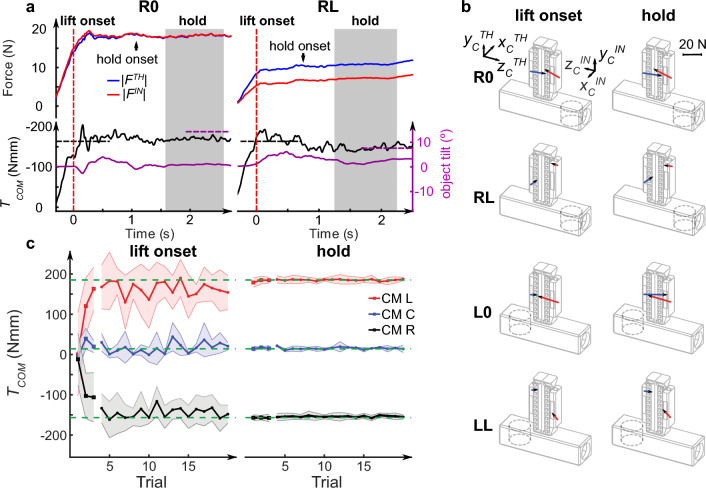
Performance of dexterous manipulation task. Data are from trials of a representative subject (S10) after learning of the manipulation task had occurred (trial 4). (**a**) Time courses of the magnitudes of digit contact forces (|*F*|, top panel) exerted by the thumb and index finger (*TH* and *IN*, blue and red lines, respectively). The bottom panel shows the time course of compensatory torque (*T*_*COM*_) and object tilt angle (black and purple lines, respectively). The black horizontal dashed lines denote the negative external torque caused by the added mass to the right side of the object’s base (for graphical purposes, the negative external torque is plotted with the same sign as *T*_*COM*_). The purple horizontal dashed lines denote peak object tilt measured on the first lift. The red vertical dashed lines and gray areas denote object lift onset and the hold epoch used for analysis, respectively. Data are from the right center of mass condition at zero and large digit offsets (R0 and RL conditions, left and right column, respectively). (**b**) Three-dimensional force vectors at individual digit contacts for the same trials in R0 and RL at lift onset (left column) and hold phase (right column), and from the left center of mass condition (L) at zero and large digit offsets (L0 and LL, respectively). (**c**) Compensatory torque at object lift onset and during the hold phase from each trial averaged across all digit offset conditions (shaded areas denote ± S.D.) from the same subject shown in (**a**) and (**b**). The green horizontal dashed lines denote the external torque (*T*_*EXT*_) caused by the added mass (L or R center of mass, CM; C denotes center CM).

To minimize object tilt, subjects must learn to generate a compensatory torque (*T*_*COM*_) at object lift onset to counter the external torque (*T*_*EXT*_) caused by the mass added to the left or right side of the object’s base (Fig. 1b). *T*_*COM*_ was computed according to the Eq. (6) (see Sect. “Data analysis” in "Materials and methods"). At the same time, subjects must exert digit forces to prevent object slip during manipulation. All subjects quickly learned our dexterous manipulation task, as indicated by the attainment of a stable *T*_*COM*_ after the third trial (no significant effect of *Trial* after removing the first three trials for each condition; t_17.51_ = 1.97, *p* = 0.065). Figure 2a shows kinetic data from two trials (trial 4) from right center of mass (CM) conditions performed at zero and large digit offsets (R0 and RL, respectively) by a representative subject. The top row of Fig. 2a depicts time courses of the magnitude of the contact forces vector from thumb and index finger (*F*^*TH*^ and *F*^*IN*^, respectively), whereas the bottom row shows time courses of *T*_*COM*_ and object tilt angle. After contacting the object with both digits, *T*_*COM*_ approached *T*_*EXT*_ around object lift onset, resulting in small deviations from the object’s vertical orientation in both experimental conditions (~ ± 5°).

Figure 2b illustrates the three-dimensional digit force vectors at the two epochs we used for analyses (object lift onset and hold; left and right column, respectively). The direction of the force vectors appears to remain constant from lift onset to object hold while their magnitude is modulated according to the experimental condition. This phenomenon suggests that digit force coordination had already been established before the onset of manipulation, i.e., object lift, to be fine-tuned throughout object lift and hold. However, our experimental manipulation of digit offset caused distinct patterns of digit force coordination. Specifically, the magnitude of the thumb and index finger force vectors was greater for zero-digit offsets than large digit offsets at object hold (R0 and L0 versus RL and LL, respectively), this difference being smaller at object lift onset.

Subjects quickly learned to generate the required *T*_*COM*_ at object lift onset and during the hold phase. After the third trial in each condition block, *T*_*COM*_ was slightly lower than *T*_*EXT*_ at lift onset, but matched it more closely during object hold (Fig. 2c and Supplementary Fig. S1). Task performance was evaluated by computing the resultant torque, *T*_*RES*_, as the difference between *T*_*EXT*_ and *T*_*COM*_, where a value of zero denotes optimal task performance (0° object tilt). The observations of the trial-to-trial changes in performance from the representative subject in Fig. 2 were confirmed by analyses of data from all subjects using three-way LMMs on *T*_*RES*_ with factors *CM*, *Digit Offset* and *Trial* at lift onset and object hold. At lift onset, subjects learned to exert *T*_*COM*_ after the third trial for both CM conditions (significant coefficients: − 79.83 N·mm, t_59.93_ =  − 9.86, *p* < 0.0001 and 82.13 N·mm, t_15.57_ = 7.15, *p* < 0.0001 for the left and right CM, respectively). During the hold phase, *T*_*RES*_ was close to zero for all conditions (t_9.07_ =  − 1.85, *p* = 0.0968).

### Grasp and manipulation forces are concurrently modulated as a function of grasp configuration

**Figure 3:**
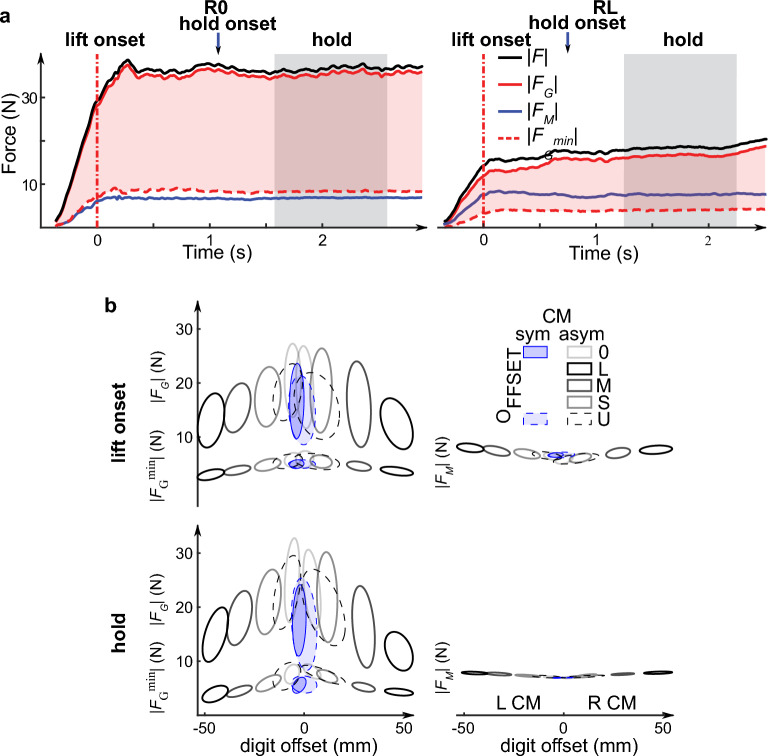
Grasp and manipulation forces. (**a**) Time courses of the magnitude of total digit force (*F*), grasp force (*F*_*G*_), manipulation force (*F*_*M*_) and minimum grasp force vectors required to prevent object slip (*F*_*G*_^*min*^) for the same trials and experimental conditions performed by the representative subject shown in Fig. 2. Pink shaded areas denote *F*_*G*_ above *F*_*G*_^*min*^. Red vertical dashed lines and gray areas indicate object lift onset and the time window within the hold phase used for analysis, respectively. (**b**) Data clusters of grasp force and minimum grasp force (left column), and manipulation force (right column) are plotted for each experimental condition as ellipses measured at object lift onset and hold (top and bottom row, respectively). The ellipses were computed using data from trials 4–20 and all subjects in each digit offset condition. The length of the principal axes of each ellipse was computed using principal component analysis. The half-length of each of the two principal axes denote the standard deviation along corresponding axes.

To understand the force coordination strategies used by subjects to simultaneously prevent object slip and control object pose, we decomposed digit forces and moments into grasp (*F*_*G*_) and manipulation (*F*_*M*_) forces (see Analysis of grasp and manipulation forces in Sect. "Materials and methods"). Figure 3a shows the time course of the magnitude of total digit force (*F*), *F*_*G*_ and *F*_*M*_ vectors, together with the magnitude of the minimum grasp force vector required to prevent object slip (*F*_*G*_^*min*^) from the same subject and experimental conditions shown in Fig. 2a. *F*_*G*_ and *F*_*M*_ increased in parallel from contact and throughout the manipulation, although the rate at which *F*_*G*_ increased was greater than *F*_*M*_. Also, the difference between *F*_*G*_ and *F*_*M*_ was much greater for the zero than large digit offset (R0 and RL, respectively)*.* However, regardless of digit offset, this subject exerted two-fold larger *F*_*G*_ than *F*_*G*_^*min*^.

The patterns described above for one subject were common to all subjects. Figure 3b plots the magnitude of *F*_*G*_, *F*_*M*_ and *F*_*G*_^*min*^ as data clusters for each experimental condition as an ellipse measured at object lift onset and hold (top and bottom row, respectively). As the measured digit offset in each condition was not identical across trials even within the same subject, for graphical purposes we plotted the data as ellipses to illustrate across-trial and -subject variability of digit offsets (horizontal axis) and forces (vertical axis). Each ellipse was computed using principal components analysis applied on data from trials 4–20 and all subjects in each digit offset condition (0, L, M, S, and U; see Sect. "Materials and Methods"). With increasing digit offset, subjects exerted smaller* F*_*G*_ (t_15.03_ =  − 8.47, *p* < 0.0001 and t_21.52_ =  − 10.50, *p* < 0.0001 for lift onset and hold, respectively; left column, Fig. 3b). We also found that *F*_*G*_^*min*^ decreased with increasing digit offset (t_13.48_ =  − 18.18, *p* < 0.0001 and t_17.99_ =  − 9.58, *p* < 0.0001 for object lift onset and hold, respectively; left column, Fig. 3b). Subjects exerted larger *F*_*M*_ with increasing digit offset, i.e., an opposite relation with digit offset compared to *F*_*G*_ and *F*_*G*_^*min*^ (t_15.88_ = 14.29, *p* < 0.0001 and t_15.00_ = 15.52, *p* < 0.0001 for lift onset and hold, respectively; right column, Fig. 3b).

In sum, we found opposite effects of increasing digit offset on digit forces, such that *F*_*G*_ and *F*_*G*_^*min*^ decreased whereas *F*_*M*_ increased (Fig. 3b). Although the modulation of *F*_*G*_, *F*_*M*_ and *F*_*G*_^*min*^ in the U condition exhibited the same trend as other digit offset conditions, it overlapped the modulation associated with small digit offsets. The following analyses address the extent to which these results stemmed from biomechanically-obligatory constraints versus control mechanisms.

### The modulation of manipulation force to digit offset is not caused by biomechanical constraints

*F*_*M*_ is responsible for two components of object manipulation or pose control: changes in position (accelerating the object mass vertically during the lift) and ensuring that the orientation of the object remains vertical throughout object lift and hold (countering the external torque). The contribution of *F*_*M*_ to changing object position remains invariant across digit offsets (object mass is constant). In contrast, increasing digit offset causes a biomechanically-obligatory change in the contribution of the normal and tangential components of *F*_*M*_ (^*z*^*F*_*M*_ and ^*y*^*F*_*M*_, respectively) to the control of object orientation. This is because the moment arm of ^*z*^*F*_*M*_ increases with larger digit offset. In contrast, the moment arm of ^*y*^*F*_*M*_ (object width; see Eq. (6)) is constant. Note that the change in ^*z*^*F*_*M*_ and ^*y*^*F*_*M*_ contributions does not necessarily imply a change in the magnitude of the *F*_*M*_ vector. Therefore, to gain insight into why subjects generated larger *F*_*M*_ with increasing digit offset, we analyzed the relation between ^*y*^*F*_*M*_ and ^*z*^*F*_*M*_ from object lift onset to hold.

**Figure 4:**
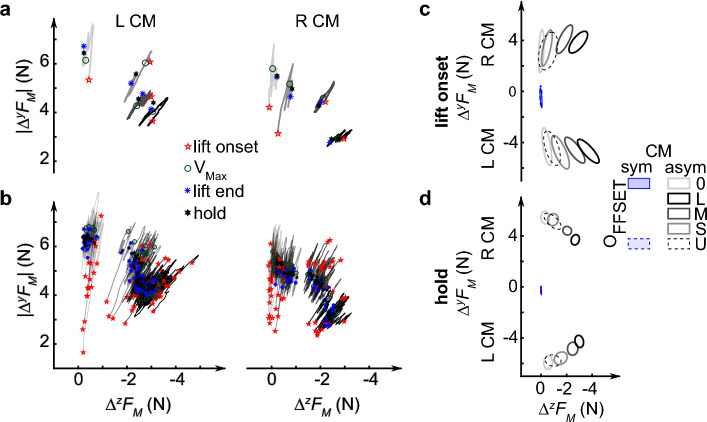
Coordination of normal and tangential components of manipulation force. (**a**) Difference of thumb and index finger tangential and normal components of manipulation force (*∆*^*y*^*F*_*M*_ and *∆*^*z*^*F*_*M*_, respectively) evolved with time in one representative trial (trial 4) of each condition from the representative subject shown in Fig. 2*.* (**b**) The coordination of *∆*^*y*^*F*_*M*_ and *∆*^*z*^*F*_*M*_ evolved with time in trials 4–20 for individual digit offset from the representative subject. (**c** and **d**) Relation between *∆*^*y*^*F*_*M*_ and *∆*^*z*^*F*_*M*_ for each experimental condition at object lift onset (**c**) and during hold (**d**). Data in (**c**) and (**d**) (trials 4–20, all subjects) are shown in the same format as Fig. 3b.

We first computed the difference between thumb and index finger *F*_*M*_ components (*∆*^*y*^*F*_*M*_ and *∆*^*z*^*F*_*M*_) to focus on object orientation control (see Sect. "Materials and Methods"). Figure 4a shows the temporal evolution of the two *F*_*M*_ components from object lift onset to object hold from one representative trial for each digit offset and object CM, whereas Fig. 4b shows data from trials 4–20 performed by the same representative subject shown in Fig. 2. As noted above, this subject had to change the contribution of each *F*_*M*_ component as a function of digit offset: at the smallest digit offset (smaller moment arm for *∆*^*z*^*F*_*M*_), the contribution of *∆*^*y*^*F*_*M*_ is much larger than *∆*^*z*^*F*_*M*_, whereas a more even contribution is found for larger digit offsets. Further examination of these trajectories from all trials (Fig. 4b) reveals two phenomena: (1) at object lift onset, *∆*^*y*^*F*_*M*_ is characterized by much greater across-trial variability than *∆*^*z*^*F*_*M*_ and (2) the coordination pattern exhibited during object hold is attained much earlier, i.e., at the time of peak object lift velocity (average across all digit offsets: 435 ± 103 ms from object lift onset).

The effects of digit offset on the coordination between *F*_*M*_ components and their temporal evolution described for the representative subject were common to all subjects (Fig. 4c,d). Figure 4d shows that the two *F*_*M*_ components covaried negatively during object hold (correlation coefficient, *r*: − 0.61, t_15.07_ =  − 12.64, *p* < 0.0001) and that *∆*^*y*^*F*_*M*_ decreased with increasing digit offset (t_15.54_ =  − 17.32, *p* < 0.0001). In contrast, at object lift onset the two *F*_*M*_ components covaried positively at lift onset (*r*: 0.29, t_14.72_ = 5.19, *p* = 0.0001) and *∆*^*y*^*F*_*M*_ significantly increased with increasing digit offset (t_15.13_ = 2.14, *p* = 0.0486) (Fig. 4c). Again, the modulation of *∆*^*z*^*F*_*M*_ and *∆*^*y*^*F*_*M*_ in U condition overlapped the modulation associated with small digit offsets.

The above analysis revealed that the significant increase in *F*_*M*_ magnitude with increasing digit offset (Fig. 3b) was caused by a parallel increase and decrease in *∆*^*z*^*F*_*M*_ and *∆*^*y*^*F*_*M*_, respectively (Fig. 4d). If subjects had prioritized exerting a constant *F*_*M*_ regardless of grasp configuration, they could have achieved this goal by re-distributing the two *F*_*M*_ components accordingly. However, our data indicate that the biomechanically non-obligatory increase in *F*_*M*_ magnitude occurred because of the stronger contribution of larger *∆*^*z*^*F*_*M*_ exerted at increasing moment arms (i.e., digit offset) than smaller *∆*^*y*^*F*_*M*_ exerted at a constant moment arm.

### The modulation of grasp force to digit offset is not only caused by biomechanical constraints

*F*_*G*_ is responsible for preventing object slip throughout object manipulation. We found that both *F*_*G*_ and *F*_*G*_^*min*^ decreased with increasing digit offset (Fig. 3b). *F*_*G*_^*min*^ was computed by optimizing biomechanical constraints, including the coefficient of friction and *F*_*M*_ (see Sect. "Materials and Methods"), and therefore its modulation with grasp configuration is biomechanically obligatory to prevent object slip. In contrast, *F*_*G*_ magnitude above *F*_*G*_^*min*^, i.e., excessive grasp force (*F*_*G*_^*EX*^), is not biomechanically obligatory. To address whether subjects modulated *F*_*G*_^*EX*^ to digit offset, we first normalized it relative to *F*_*G,*_ yielding our version of relative grasp safety margin, *SM*_*G*_ (see Sect. "Materials and Methods"). When *SM*_*G*_ is 0, *F*_*G*_ is equal to *F*_*G*_^*min*^, whereas *SM*_*G*_ approaching a value of 1 denotes that *F*_*G*_ consists primarily of *F*_*G*_^*EX*^. We note that *SM*_*G*_ captures the CNS’ response to changes in digit offset more accurately than *F*_*G*_ because it isolates the non-mechanically obligatory portion of *F*_*G*_, i.e., the grasp force that participants chose to prevent object slip.

We found that *SM*_*G*_ was positively related to digit offset at lift onset for both CMs, and during hold for the left CM (lift onset: coefficient = 0.0014, t_15.10_ = 3.71, *p* = 0.002 for both right and left CM; hold: coefficient = 0.00007, t_15.12_ = 0.21, *p* = 0.84 and coefficient = 0.0019, t_17.74_ = 5.06, *p* < 0.0001 for right and left CM, respectively; Supplementary Figure S2), i.e., an opposite relation relative to the above-described relation between *F*_*G*_ and digit offset (see Fig. 3b, left column). When examining the variables used to compute *SM*_*G*_, an important finding was that *F*_*G*_ and *F*_*G*_^*min*^ were differentially affected by increasing digit offset. Specifically, *F*_*G*_ decreased at a slower rate than *F*_*G*_^*min*^ as indicated by the significant decrease in the ratio between *F*_*G*_^*min*^ and *F*_*G*_ with increasing digit offset for lift onset (both CM) and hold (left CM) (slope: − 0.0014 for both right and left CM at lift onset; slope: − 0.0019 for left CM during hold).

Importantly, the biomechanically-obligatory component of *SM*_*G*_ (*F*_*G*_^*min*^) decreased at a higher rate with digit offset than the combination of obligatory and non-obligatory components (*F*_*G*_), as indicated by the significant decrease in their ratio except for the right CM at hold (both CM at lift onset: t_15.29_ =  − 3.74, *p* = 0.0019; t_15.12_ =  − 0.21, *p* = 0.837 and t_15.59_ =  − 4.86, *p* = 0.0002 for right and left CM during hold, respectively). Further analysis of normal and tangential components of *F*_*G*_^*EX*^ (^*z*^*F*_*G*_^*EX*^ and ^*y*^*F*_*G*_^*EX*^) revealed that the disproportionate increase of *F*_*G*_ relative to *F*_*G*_^*min*^ was primarily caused by ^*y*^*F*_*G*_^*EX*^ (for details see S3. Coordination between *F*_*M*_ and *F*_*G*_).

The results discussed so far have dealt with separating biomechanically-obligatory from non-obligatory digit force response to changes in digit offset. In the next section, we address the putative sensorimotor control mechanisms responsible for planning and execution of *F*_*M*_ and* F*_*G*_.

### Planning and execution of digit force coordination

To address the extent to which execution of *F*_*M*_ and* F*_*G*_ is mediated by anticipatory mechanisms or also involve feedback-based mechanisms, we analyzed the above-described non-obligatory modulation of *F*_*M*_ and *F*_*G*_^*EX*^ by first comparing these forces at object lift onset versus hold. *F*_*G*_ magnitude above *F*_*G*_^*min*^, i.e., excessive grasp force (*F*_*G*_^*EX*^), was the same at these two episodes (t_15.00_ = -2.13, *p* = 0.05). In contrast, *F*_*M*_ was significantly larger during object hold than at lift onset (t_14.97_ = -10.4, *p* < 0.0001).

**Figure 5:**
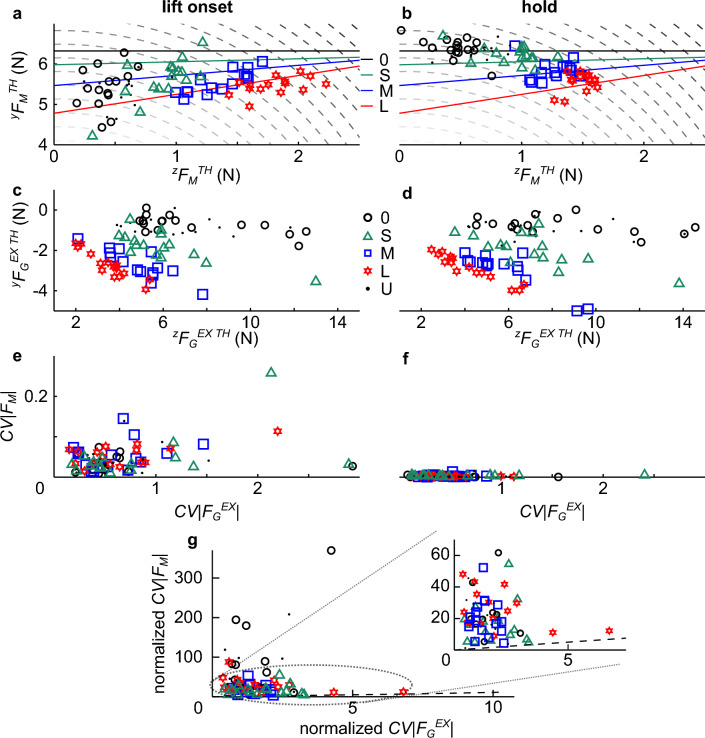
*F*_*M*_ and *F*_*G*_ components and coefficient of variations. F_M_ component data (simulated solution space and experimental data) from object lift onset and object hold (**a** and **b**, respectively) are plotted on the ^y^*F*_*M*_ and ^z^*F*_*M*_ plane of thumb digit forces. Solid lines denote the solution space that satisfy task constraints (Eqs. (4–6), Material and Methods) for the desired *T*_*COM*_ at each digit offset (0, S, M, and L). Combinations of *F*_*M*_ components along the solid lines are equally valid for optimal control of object pose (0° tilt) within a given digit offset condition. The dashed quadratic lines denote *F*_*M*_ component combinations that result in the same *F*_*M*_ magnitude summed across thumb and index finger (|*F*_*M*_| in Fig. 3b). Larger *F*_*M*_ magnitudes are denoted by darker quadratic lines. *F*_*G*_ component data (experimental data) from object lift onset and object hold (**c** and (**d**), respectively) are plotted on the ^y^*F*_*G*_ and ^z^*F*_*G*_ plane of thumb digit forces. Each experimental data point in (**a**–**c**), and (**d**) is the average of trials 4–20 within each digit offset condition (0, S, M, L, and U) from individual subjects from the L CM condition. **e** and (**f**) show the coefficient of variations (*CV*s) of *F*_*M*_* and F*_*G*_ at object lift onset and object hold. Each data point in **e** and **f** is the *CV* computed from trials 4–20 within each digit offset condition (0, S, M, L, and U) from individual subjects from the L CM condition. (**g**) shows *CV*s at object lift onset normalized to *CV*s during hold. The inset shows the area of the plot where most data clustered. Dashed lines denote unity lines.

Further analysis showed that the re-distribution of *F*_*M*_ components, although indeterminate, obeyed the constraints of manipulation dynamics (solid lines in Fig. 5a,b), e.g., object lift, hold and orientation control. However, *F*_*M*_ components, and in particular ^*y*^*F*_*M*_, underwent major re-distribution from object lift onset to static hold (Fig. 5a and b, respectively). In contrast, the modulation of *F*_*G*_ components above minimally-required *F*_*G*_ to prevent object slip was negligible from object lift onset to hold (Fig. 5c and d, respectively). These observations were supported by the greater across-trial coefficient of variation for *F*_*M*_ (*CV|F*_*M*_*|*) computed at object lift onset than during hold (Fig. 5e,f). In contrast, the *CV* for excessive *F*_*G*_ (*CV|F*_*G*_^*EX*^*|*) remained approximately invariant at these two time epochs.

The statistical comparison of *CV*s at object lift onset versus hold confirmed that *CV|F*_*M*_*|* was significantly larger at object lift onset than hold (t_21.13_ = 10.10, *p* < 0.0001), whereas *CV|F*_*G*_^*EX*^*|* was not significantly different between these time epochs (t_17.41_ = 2.59, *p* = 0.019). In order to visualize the contrast of changes from object lift onset to static hold between *CV|F*_*M*_*|* and *CV|F*_*G*_^*EX*^*|,* Fig. 5g shows *CV|F*_*M*_*|* and *CV|F*_*G*_^*EX*^*|* at object lift onset normalized to their respective *CV* during hold. This figure clearly shows that normalized across-trial variability of *F*_*M*_ at object lift onset was consistently larger than *F*_*G*_^*EX*^ across all digit offsets, all values being clustered above the unity line (range of *CV|F*_*G*_^*EX*^*|*: 0.38 to 6.81 ; range of *CV|F*_*M*_*|*: 1.27 to 369.58). Therefore, subjects consistently exerted similar *F*_*G*_^*EX*^ from object lift onset to the end of manipulation. In contrast, *F*_*M*_ underwent significant and variable modulation across the two time epochs.

These findings suggest that *F*_*G*_^*EX*^ could be planned and executed in a feedforward fashion, whereas the acquisition of somatosensory feedback after object lift onset contributed to modulating *F*_*M*_ throughout the dynamic phase of manipulation.

## Discussion

By decomposing digit forces into grasp and manipulation forces, our application of robotic grasp analysis to human digit forces revealed that modulation of digit forces for manipulation and grasp involves distinct anticipatory and feedback-based mechanisms, respectively. Specifically, subjects planned *F*_*G*_ at object lift onset as a function of digit offset and exerted the same grasp force throughout object lift and during object hold. In contrast and contrary to our hypothesis, manipulation force exerted at object lift onset did not accurately predict the consequences of hand-object interaction (Figs. 4b and 5a), suggesting that subjects relied on somatosensory and visual feedback to modulate manipulation force following object lift onset. Digit forces modulation in the U condition exhibited the same trend as all the other digit offset condition and overlapped the modulation associated with small digit offsets. Below we discuss these insights and putative biological control mechanisms.

### Chain of effects caused by changes of grasp configuration on the coordination of grasp and manipulation forces

**Figure 6:**
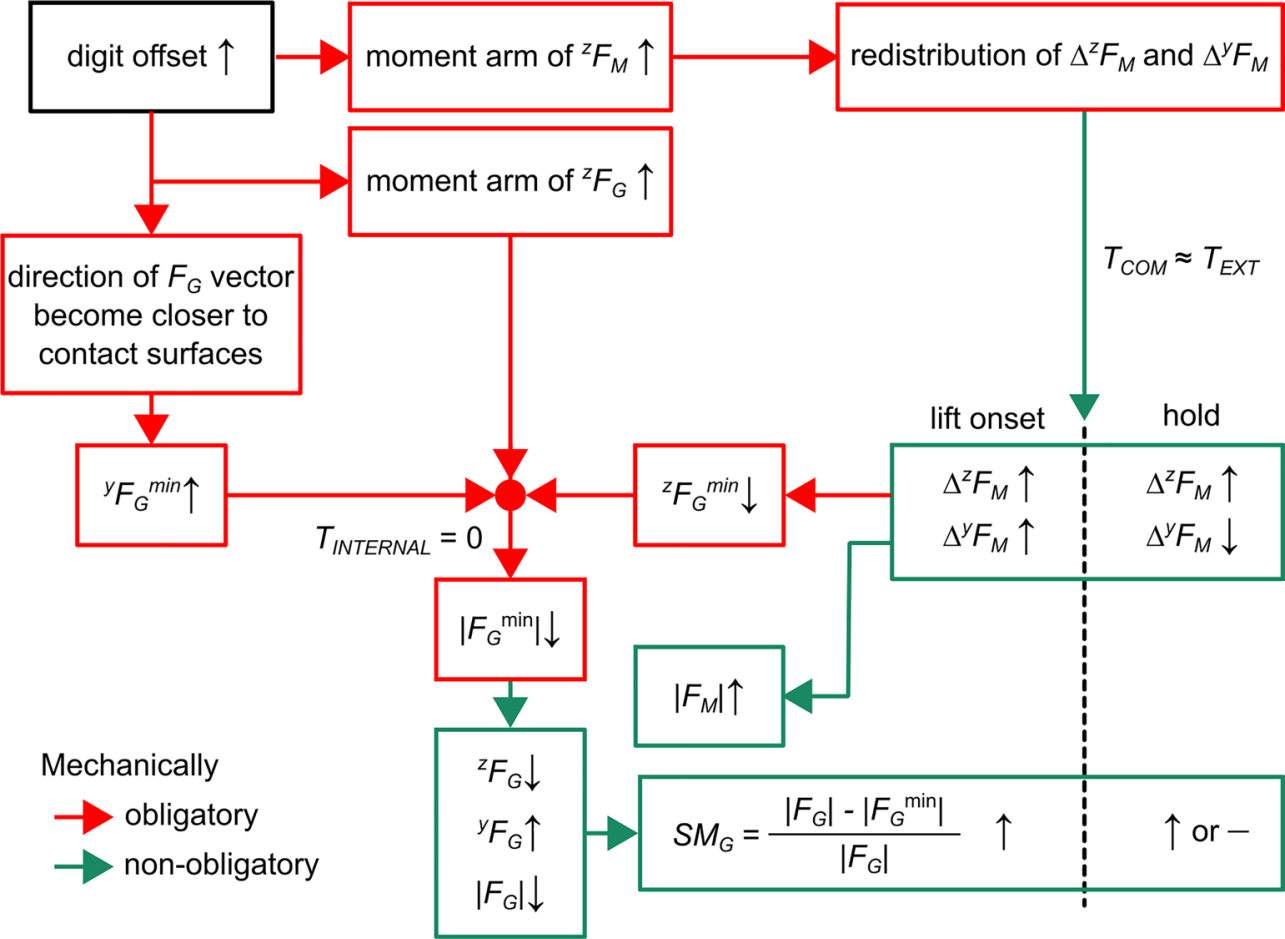
Chain of effects caused by changes in grasp configuration on the coordination of *F*_*G*_ and *F*_*M*_. The diagram summarizes the chain of effects caused by changes in grasp configuration. The chain of effects starts from increasing digit offset and ends with an increase and decrease in *F*_*M*_ and *F*_*G*_, respectively, culminating with a larger or constant *SM*_*G*_ at object lift onset and hold. Upward and downward arrows denote an increase or decrease of a given variable, whereas a horizontal line denotes no change. Red and green lines denote biomechanically-obligatory and non-obligatory effects, respectively. *T*_*COM*_ denotes the compensatory torque *F*_*M*_ contributes to and that minimizes object tilt by countering the external torque (*T*_*EXT*_) caused by the mass added to the left or right side of the object’s base. T_INTERNAL_ denotes the zero net torque generated by *F*_*G*_ exerted by each digit.

We expected changes in grasp configuration to cause multiple changes in digit force coordination. The decomposition of *F*_*M*_ from *F*_*G*_ was instrumental in separating effects due to biomechanical factors from control strategies chosen by the subjects to prevent object slip and control object pose as required by our task. Figure 6 summarizes the results of our analyses and describes how changing grasp configuration created a chain of biomechanically-obligatory and non-obligatory effects on the modulation of digit forces (red and green lines/boxes, respectively; Fig. 6).

With regard to *F*_*M*_, increasing digit offset resulted in an increase in the moment arm of the *F*_*M*_ normal component, causing a biomechanically-obligatory re-distribution of normal and tangential *F*_*M*_ components (*∆*^*z*^*F*_*M*_ and *∆*^*y*^*F*_*M*_, respectively). However, as the distribution of these components is mathematically indeterminate (Eq. (6)), subjects modulated *∆*^*y*^*F*_*M*_ and *∆*^*z*^*F*_*M*_ at object lift onset differently relative to hold (Fig. 4). The modulation of these *F*_*M*_ components resulted in an obligatory decrease in ^z^*F*_*G*_^*min*^ (the normal force required to prevent slip induced by manipulation). The combination of the decrease in ^z^*F*_*G*_^*min*^, the increase in its moment arm and the change in direction of the *F*_*G*_ vector to satisfy the zero *T*_*INTERNAL*_ constraint led a reduction in *F*_*G*_^*min*^ magnitude. The net result of this response was a biomechanically non-obligatory increase in *F*_*M*_ (Figs. 3b, 6) and a concurrent obligatory decrease in ^z^*F*_*G*_^*min*^ (Fig. 6).

Regarding *F*_*G*_, increasing digit offset resulted in two phenomena: an increase in the moment arm of the *F*_*G*_ normal component (^z^*F*_*G*_) and the direction of the *F*_*G*_ vector approaching the edge of the friction cone (Supplementary Fig. S3a). The latter phenomenon caused a biomechanically-obligatory increase in the minimally-required tangential component of *F*_*G*_ (^*y*^*F*_*G*_^*min*^) (Supplementary Fig. S3a). The net result of larger ^*z*^*F*_*G*_ moment arm, larger ^*y*^*F*_*G*_^*min*^ and smaller ^z^*F*_*G*_^*min*^ resulted in a decrease in *F*_*G*_^*min*^. Subjects responded to these effects by decreasing *F*_*G*_ with increasing digit offset (Fig. 3b, 6). Further analysis, however, showed that increasing digit offset caused *F*_*G*_ to decrease at a slower rate than *F*_*G*_^*min*^ which, combined with a larger *F*_*M*_, led to larger relative grasp safety margin (*SM*_*G*_).

### Biological control strategies for grasp and manipulation forces

The net consequences of the interaction among biomechanically-obligatory and non-obligatory effects caused by increasing digit offset were (1) an increase in manipulation force, (2) a decrease in grasp force and (3) an increase in relative safety margin (Fig. 6). Below we discuss possible mechanisms that might have led to the opposite modulation of grasp and manipulation forces and the extent to which this phenomenon might reflect a causal relation between these two forces.

The re-distribution of *F*_*M*_ components, although indeterminate, must obey the constraints of manipulation dynamics, e.g., object lift, hold and orientation control, which are described by six equations (object translation and rotation in three dimensions). Increasing digit offset caused an increase in ^*z*^*F*_*M*_ moment arm (Fig. 6). We note that, even for the largest digit offset, the ^*z*^*F*_*M*_ moment arm (55 mm) is still smaller than the ^*y*^*F*_*M*_ moment arm (59 mm). If subjects had prioritized minimizing *F*_*M*_ magnitude (i.e., quadratic lines closer to the origin of plot in Fig. 5ab) to minimize energy expenditure or effort for object pose control, this goal could have been accomplished by leveraging the larger ^*y*^*F*_*M*_ moment arm. However, subjects generated greater *F*_*M*_ with larger digit offsets, which was mostly caused by larger ^*z*^*F*_*M*_ (Fig. 4d). There might be a functional benefit resulting from this apparent inefficient re-distribution in *F*_*M*_ components. Although the mechanical effects of digit forces on object slip prevention and object pose control can be mathematically decomposed into *F*_*G*_ and *F*_*M*_, respectively, modulation of *F*_*M*_ components impacts the risk of object slip differently: larger ^*y*^*F*_*M*_ directly increases the risk of object slip, whereas larger ^*z*^*F*_*M*_ does not. At the same time, for the *T*_*COM*_ and grasp configuration we used, relying on larger ^*z*^*F*_*M*_ to control object pose instead of ^*y*^*F*_*M*_ does reduce *F*_*G*_^*min*^ as less grasp force is required to prevent object slip (see *Estimation of biomechanically-obligatory and non-obligatory portions of grasp force* in Sect. "Materials and Methods"). Therefore, as the distribution of *F*_*M*_ components (^*z*^*F*_*M*_ vs. ^*y*^*F*_*M*_) is indeterminate (Fig. 5a), subjects might have preferred a strategy that minimizes its impact on object slip over effort minimization. Another consequence of the observed re-distribution of *F*_*M*_ components with larger digit offsets was a reduction in ^*z*^*F*_*G*_^*min*^, which prompted subjects to implement the non-obligatory reduction in ^*z*^*F*_*G*_ (Fig. 6).

Regarding grasp force, larger digit offsets cause the *F*_*G*_ vector to rotate and approach to edge of the friction cone (Supplementary Fig. S3a). This effect, combined with the decrease in ^*z*^*F*_*G*_^*min*^ and the increase in ^*z*^*F*_*G*_ moment arm, caused a decrease in *F*_*G*_^*min*^ (Fig. 6). Recall that, by virtue of the robotic grasp analysis technique, modulation of *F*_*G*_ does not impact object pose control, hence the internal torque, T_INTERNAL,_ is zero (Fig. 6). To gain further insight into the modulation of *F*_*G*_ as a function of digit offset, we combined *F*_*G*_ and *F*_*G*_^*min*^ to derive a version of the classic safety margin metric developed by Westling and Johansson^2^, the relative grasp safety margin, *SM*_***G***_. We found that *F*_*G*_ and *F*_*G*_^*min*^ both decreased with increasing digit offset, but did so at different rates (Supplementary Fig. S2). This finding implies that subjects were able to perceive the decrease in *F*_*G*_^*min*^, hence a reduced need to exert large *F*_*G*_ to prevent object slip. Nevertheless, they exerted larger than necessary *F*_*G*_, which could have been caused by inaccurate sensing of the rate at which *F*_*G*_^*min*^ decreased with digit offset.

If efficiency of digit force control is defined in the context of minimization of energy expenditure and fatigue, the use of greater excessive *F*_*G*_ could be viewed as inefficient. However, excessive *F*_*G*_ also increases grasp stability, defined as the system’s ability to resist perturbations. We note that excessive *F*_*G*_ can only contribute to object stability, defined within the task criterion of object slip prevention, but cannot contribute to stability of object pose control. As subjects were not exposed to external perturbations, the use of excessive *F*_*G*_ more likely reflects a strategy to counter sensorimotor noise and motor execution variability. If so, we could view the seemingly inefficient use of large *F*_*G*_ as a means to maximize grasp stability to overcome this internal source of variability. Additionally, it is conceivable that sensing of the larger tangential component of *F*_*M*_, *∆*^*y*^*F*_*M*_, at object lift onset might have prompted subjects to use a greater *SM*_***G***_ to minimize the risk of object slip at the onset of the dynamic task component.

### Anticipatory versus feedback-based control of grasp and manipulation force

Object lift onset marks an important transition across task epochs that begins with object contact and culminates with the development of digit force distributions to start manipulation. Importantly, the digit force distribution used on a given trial is controlled in an anticipatory fashion and is therefore driven by sensorimotor memory of digit forces used in previous manipulations^2,12^. This reliance on memory is critically important because feedback about object mass distribution and the effects of a given digit force distribution on object pose become available only shortly after the object starts to be accelerated^6,22,17^. If the digit force distribution attained at object lift onset is appropriate for the planned manipulation, expected and actual sensory feedback—tactile, visual and proprioceptive—acquired throughout object lift will coincide^13,23^. Conversely, if a mismatch occurs, sensory feedback will trigger digit force adjustments to correct for the motor error^11,13^.

We found that subjects modulated *F*_*G*_ to grasp configuration at object lift onset and that this modulation was maintained throughout object lift and during hold (Fig. 5c,d). This finding, which supports our first hypothesis, is consistent with and extends previous work on anticipatory modulation of grip force to object properties^11^. This can be considered evidence for subjects’s ability to build and retrieve sensorimotor memory built through previous manipulations. In contrast and contrary to our second hypothesis, *F*_*M*_ at object lift onset underwent significant modulation throughout object lift (Fig. 5a,b). We interpret this finding as indicative of subjects’ inability to build a complete model about object dynamics experience in previous manipulations and/or use it at object lift onset. Alternatively, even if subjects could have learned a perfect model of hand-object interactions, object dynamics would still be sensitive to *F*_*M*_ prediction errors and sensorimotor noise. Therefore, our results suggest that subjects modified *F*_*M*_ throughout the dynamic portion of the task by relying on sensory feedback acquired after object lift onset (Figs. 4b and 5a).

Further evidence for different sensorimotor control mechanisms of *F*_*M*_ and *F*_*G*_ was provided by analysis of their trial-to-trial variability (coefficient of variation, *CV*) for each experimental condition. Regarding *F*_*G*_, *CV* was statistically indistinguishable at object lift onset and hold (Fig. 5e,f). Therefore, although subjects used excessive *F*_*G*_, they used an anticipatory strategy throughout the task. This finding suggests that subjects prioritized grasp stability than efficiency by using a consistent control strategy. In contrast, *F*_*M*_* CV* was much greater at object lift onset than hold, again indicating more uncertainty in anticipatory control of *F*_*M*_.

In sum, the contrast between object lift onset and hold suggests that *F*_*G*_ is mediated by anticipatory control mechanisms at the onset of manipulation, whereas *F*_*M*_ requires additional somatosensory and visual feedback acquired after object lift. These differences may reflect the extent to which their modulation impacts object slip prevention versus pose control. For *F*_*G*_, one to two successful lifts without the object slipping are known to be sufficient to build a sensorimotor memory to generate stereotypical digit forces^11^. Therefore, subjects would not need online feedback during the lift to modify *F*_*G*_ used at object lift onset, unless errors occur during manipulation. In contrast, successful object pose control imposes harder constraints on *F*_*M*_ because it must satisfy force and torque equilibria requirements. Additionally, it is possible that the modulation of *F*_*M*_ and *F*_*G*_ might be mediated by different sensorimotor mechanisms. This proposition is consistent with the results of one of our recent studies showing that distinct adaption rates for internal and manipulation moments (equivalent to *F*_*G*_ and *F*_*M*_, respectively) during virtual object manipulation and the modulation of internal moments to maximize grasp stability^24^. We discuss putative neural mechanisms underlying the coordination of *F*_*M*_ and *F*_*G*_ in the next section.

### Neural mechanisms underlying the coordination of grasp and manipulation forces

The mathematical decoupling of grasp and manipulation forces we adopted from robotics raises important questions about biological sensorimotor control mechanisms. Do grasp and manipulation forces generate distinct tactile inputs from skin mechanoreceptors during manipulation? If so, how and at what level of the CNS are these distinct channels of information integrated for the coordination of grasp and manipulation forces? Conversely, if tactile inputs arising from manipulation cannot discriminate grasp from manipulation forces, how does the CNS decode this sensory information to coordinate these two forces?

Our behavioral data cannot be used to answer these questions, which will require combining our methodological approach—robotic grasp analysis and a dexterous manipulation task—with electrophysiological recordings. Nevertheless, we speculate that tactile inputs might be capable of separately encoding grasp and manipulation forces. The role of tactile receptors has been extensively studied but mostly in the context of grasp control, i.e., slip prevention (^11,13^; for a recent review see^25^). It is therefore unknown how tactile inputs arising from object manipulation might be used to coordinate grasp and manipulation forces. However, it is well known that the response of different classes of tactile mechanoreceptors is tuned to the features of skin mechanical deformation (e.g., pressure, vibration, and force direction^11,26–28^). It is therefore conceivable that the distinct spatiotemporal patterns of tactile inputs associated with different grasp configurations^29^, hence digit force vector directions, might have been instrumental in signaling the digit force distribution necessary to simultaneously prevent object slip and minimize object tilt. Specifically, tactile sensing could have been instrumental in downregulating *F*_*G*_ by sensing the decreasing *F*_*G*_^*min*^ with increasing digit offsets (Figs. 3b, 6). Despite this decrease in *F*_*G*_, *SM*_*G*_ significantly increased at object lift onset, which could be explained by tactile sensing of larger ^*y*^*F*_*M*_ at object lift onset with increasing digit offset. This phenomenon would have triggered the use of larger than necessary *F*_*G*_ as an additional measure to maximize successful object pose control during the dynamic phase of manipulation.

The speculation that grasp and manipulation forces might be encoded by separate patterns of tactile inputs leads to the question of how this information might be integrated. This integration is likely to happen within cortical association areas, e.g. parietal cortex, responsible for integration of somatosensory inputs and subsequent modulation of descending commands from pre-motor and motor areas^30–32^. Although this proposition remains to be investigated, recent work using theta burst transcranial magnetic stimulation has shown a context-dependent involvement of primary sensory and cortical cortices (S1 and M1, respectively) in dexterous manipulation. When grasping occurs at constrained contacts—which enables using a sensorimotor memory-based control of digit forces^33,34^—M1 but not S1 is involved in storing and retrieving learned digit forces and positions. However, when digit position is allowed to vary across trials (unconstrained grasping corresponding to the present U condition), integrity of M1 and S1 is instrumental in ensuring digit force modulation to position^35^. Therefore, it is also conceivable that distinct patterns of tactile inputs reaching S1 might drive the modulation of digit forces that could enable coordination of grasp and manipulation forces.

Lastly, although tactile inputs can signal both object slip and errors in pose control, the latter can also be signaled by visual feedback. Therefore, differences in the modulation of *F*_*M*_ and *F*_*G*_ across grasp configurations, as well as their different control mechanisms, might also be accounted for by differences in contributions of tactile and visual inputs both in creating sensorimotor memory from previous manipulations and performing online corrections.

### Disentangling biomechanically-obligatory from non-obligatory effects of changes in grasp configuration: Traditional versus robotic grasp analysis

Most previous work focused on normal and tangential force modulation for object slip prevention but did not address how these forces may contribute to dexterous object pose control. To address this limitation, we used a task that required subjects to coordinate digit forces to simultaneously satisfy object slip prevention and pose control requirements (Fig. 1). The present task, as the task we used in our earlier work, requires coordination of fingertip forces and position of contacts to control object pose^17^. However, the key methodological difference between our previous and current work is that here we systematically changed the grasp configuration (Fig. 1c), rather than allowing subjects to choose it on a trial-to-trial basis, i.e., the Unconstrained experimental condition. This allowed the exploration of a much larger range of digit force-position relations relative to the grasp configuration that subjects may spontaneously choose to use. Had we tested only the unconstrained digit offset condition (U; Fig. 1c), we could not have thoroughly examined digit force coordination patterns due to the significantly smaller modulation (~ 40%) of digit offset relative to all other conditions (Fig. 3b).

As noted in the Sect. “Introduction”, using a dexterous manipulation task would not have been sufficient to investigate the coordination of digit forces for simultaneously preventing object slip and controlling object pose. This is because the dual role of digit forces cannot be distinguished using traditional force analysis. To overcome this barrier, we used of a robotic grasp analysis approach to decompose grasp and manipulation forces without constraining contacts to be horizontal and collinear.

Another distinctive feature of the present work is that, unlike previous work, we probed the mechanisms of digit force coordination by changing grasp configuration rather than object properties. The rationale for focusing on grasp configuration was that it allows examining the control strategies subjects select from a large space of equally-valid digit force coordination patterns. In contrast, had we kept grasp configuration invariant and a task with no dexterity component, subjects could have responded to changes in object properties, e.g., mass or contact texture, by using one control strategy. For example, grasping and manipulating an object with variable object mass or frictional properties can be performed by applying a constant safety margin to prevent object slip^2,8^.

## Conclusions

Our results provide, for the first time, a different perspective on the coordination of digit forces relative to previous research on grasping and manipulation. By going beyond the task requirement of object slip prevention and decomposing grasp from manipulation forces using robotic grasp analysis, we found that subjects prioritized grasp stability over efficiency of grasp force control and that this prioritization was maintained throughout the task. Manipulation force was modulated following acquisition of somatosensory feedback after object lift onset, underscoring challenges with accurately anticipating object dynamics. We conclude that manipulation force appears to rely on online somatosensory and visual feedback to a greater extent than grasp force.

The present work also lays the foundation for new research avenues, including the application of our approach to a broader variety of hand-object interactions and task scenarios, as well as biomedical applications, e.g., biologically-inspired grasp controllers for human–machine interfaces, neuroprosthetics and assistive devices. An open question is whether distinct neural representations of *F*_*M*_ and *F*_*G*_ can be decomposed from neuronal populations. If so, one could envision brain-machine interfaces to control prosthetic or robotic hands where object slip prevention and pose control could be modulated independently as a function of task demands. Additional critical questions that should be addressed by future research include determining (1) the extent to which the findings may generalize to other object manipulation tasks or hand-object interactions with different features, such as stricter requirements for object control, and (2) the extent to which tactile input patterns might encode grasp and manipulation forces separately and their integration.

## Materials and methods

### Experimental design

We applied digit force analysis tools designed for robotic manipulation on the forces exerted by the participants performing a dexterous task that requires simultaneous object slip prevention and tilt minimization at different grasp configurations. We asked participants to grasp an instrumented inverted-T shape object (Fig. 1a) with thumb and index fingertip, lift it while preventing it from tilting, and hold it. We systematically changed the object’s mass distribution and the vertical distance between the digits (offset; Fig. 1c). The former factor was introduced to add a dexterity component to our manipulation task. The rationale for increasing digit offset was that it changes the moment arm of normal digit forces, and therefore their contribution to manipulation.

### Participants

Sixteen healthy right-handed (self-reported) adults aged 19–38 (23.88 ± 5.50) years (8 males) with normal to corrected vision and no history of neurological disorders were recruited for participation to the study. All individuals were naïve to the purpose of this study and provided written informed consent in accordance with the Declaration of Helsinki. The experiment protocols were approved by the Office of Research Integrity and Assurance (IRB ID: STUDY00006050) at Arizona State University.

### Apparatus

We asked subjects to grasp and manipulate a custom-made grip device using the thumb and index fingertip. The inverted T-shape device consisted of a sensorized vertical handle connected to a horizontal structure (Fig. 1a). The handle has two parallel graspable surfaces (length = 80 mm; width = 24 mm; distance = 59.2 mm) covered with sandpaper (100-grit). Two light-emitting diode (LED) arrays matching the length of the graspable surfaces were placed on the frontal plane of the object to cue subjects about the location of digit placement for each experimental condition (Fig. 1b,c). The LED arrays were controlled via a microcontroller board (Arduino Uno Rev 3, Arduino, Boston, Massachusetts, USA). Each graspable surface was instrumented with one six-component force/torque (F/T) transducer (Nano 25, ATI Industrial Automation, Garner, NC, USA; nominal force resolution: 0.0625 N; nominal torque resolution: 0.076 N cm). The transducers measure forces and moments of forces exerted by each digit on the graspable surfaces (sampling rate: 1 kHz).

The center of mass (CM) of the object was configured to be symmetrical (Center CM, *C*) or asymmetrical (Left or Right CM, *L* or *R*, respectively). For each of these conditions, we placed a mass (0.3 kg) in one of the three compartments (center, left or right) of the horizontal base of the grip device (Fig. 1b, CM). The torques caused by the added mass about the *x*-axis of the handle’s center of geometry in the handle coordinates were − 0.17, 0, and + 0.17 N m when the mass was in the left, center or right compartment, respectively. The cables of the force transducers and the LED arrays ran away from the handle with a slight offset to the left of the handle center and created a small torque 0.01 N m. Therefore, the actual task torques were − 0.18, − 0.01, and 0.16 N m for CM L, C, and R, respectively. Note that the terms “left” and “right” denote the thumb and index finger side of the vertical handle, respectively. The total mass of the object was 0.7 kg.

We used five thumb-index fingertip vertical distances (Fig. 1c, offset): 0 mm (0), 8.84 mm (Small, *S*), 29.7 mm (Medium, *M*), and 55.4 mm (Large, *L*). An additional experimental condition (Unconstrained, *U*) consisted in allowing subjects to grasp the object anywhere along the vertical graspable surfaces. The rationale for testing combinations of CM and offsets is described in the *Effects of digit offset on grasp force* section.

The position and orientation of the object and hand were tracked by a ten-cameras active infrared marker motion capture system (Impulse, PhaseSpace Inc., San Leandro, CA, USA). We used four infrared LED markers on the object (Fig. 1a, black dashed circles, sampling rate: 480 Hz; spatial accuracy: ~ 1 mm; spatial resolution: 0.1 mm). On each trial, the LED arrays and an audio signal cued the participants about the upcoming task event and grasp condition (see *Experimental procedures*). A customized LabVIEW program (National Instruments, Austin, TX, USA) was used to drive the microcontroller board and stream kinetic and kinematic data to the hard drive of the host computer.

### Experimental procedures

Each participant reached and manipulated the object for a total of 12 experimental conditions. These conditions consisted of 12 combinations of three CM locations and five offsets: C0, CU, L0, LS, LM, LL, LU, R0, RS, RM, RL, and RU. These combinations of external torques and offsets were selected by taking into consideration the extent to which participant could perform the task comfortably. Note that we presented participants with opposite offsets when grasping and manipulating the object with the L or R CM. Specifically, for three L CM conditions (LS, LM and LL), the LED cued participants to place the thumb higher than the index fingertip, whereas for the R CM conditions (RS, RM and RL) subjects were required to place the thumb lower than the index fingertip. The LED arrays indicated the designated contact locations of the thumb and index fingertip by lighting up according to the offset condition (see five digit offset examples in Fig. 1c). The distance spanned by each set of three LEDs (18.84 mm) was sufficiently large to enable positioning of the tip of the thumb and index finger. Participants were instructed to position their fingertips as close as possible to the active LED arrays and avoid hitting the contact surface with fingernails. Compliance with this task requirement was verified online for each trial. In the constrained offset blocks (0, S, M, and L), three LEDs lit up on each side of the graspable surface. For the unconstrained grasping block (U), all LEDs in both arrays lit up to inform participants they could choose digit placement^17^. The unconstrained and all other constrained grasp conditions share one feature: the thumb and index force vectors are not horizontal and collinear. This feature makes these experimental conditions suitable to address one of the limitations of previous research (see Introduction). However, our previous work on unconstrained grasping has also shown that the trial-to-trial digit force modulation was too small to address how digit forces are coordinated to concurrently prevent object slip and control object pose^17,22,36,37^. Nevertheless, we included the unconstrained grasp condition in the present study to examine potential differences in control strategies relative to all the constrained grasp conditions.

Before the experiment, participants washed their hands with soap and warm water to normalize skin condition. The coefficient of friction between the fingertips and the graspable surfaces was estimated from slip force measurement as the ratio between the minimal finger force normal to the graspable surface required to prevent slip to the tangential finger force measured at the object slip onset^2^. This method requires fingertip tangential (vertical) forces to be equal and aligned with the gravitational force. Therefore, we performed the slip force measurements using the object in the symmetrical (center) CM configuration. Participants sat in front of the table and held the object with the mass added to the center slot (0 N·m torque) 5 cm above the table while following the instructions to “slowly move the index finger and thumb apart and let the object drop freely when it slips.” Participants performed three object release trials at the beginning and end of the experimental session.

To familiarize with the object, cues and task, subjects performed three CU trials. These trials were followed by 20 consecutive trials for each experimental condition (20 × 12 = 240 trials). The pseudo-randomization of the 12 experimental condition blocks was designed to present a different CM across consecutive blocks. The object was placed 30 cm in front of the participant on a leveled tabletop 30 cm below the participant’s shoulder joint. This configuration ensured subjects could perform a comfortable grasp. The first auditory cue (*Ready*) prompted the subject to prepare for the reach. After 1.5 s, the second cue from the LED arrays informed subjects where to place their digits and cued them to start the reach, grasp and lift at a self-paced, natural speed. Participants were instructed to (a) grasp the object by placing, as accurately as possible, the tip of their thumb and index finger of their right hand such that each coincided with the active LEDs while extending the other digits, (b) lift the object while preventing it from tilting, as if the object were a cup filled with liquid, and (c) hold it straight for 2 s. Fulfilling the object tilt minimization criterion required participants to plan and generate a compensatory torque (*T*_*COM*_) at object lift onset to compensate for the external torque caused by the object’s asymmetrical mass distribution (R and L CM conditions)^17^. When the object reached a height of 20 cm above the table, a third auditory cue (*Hold*) was triggered, after which subjects were required to hold the object stationary and in a vertical orientation for ~ 2 s. The next auditory cue (*Relax*) informed subjects to replace the object on the table and move the hand back to the initial position. Subjects were given ~ 10-s breaks between trials and ~ 30 s breaks between blocks.

As the CM of the object cannot be visually inferred (the added mass is hidden from view), subjects do not exert adequate *T*_*COM*_ at lift onset on the first left or right CM trial, thus causing the object to tilt during the lift. However, we previously found that subjects can learn to generate an anticipatory *T*_*COM*_ to minimize object tilt within the first three trials^17^. This phenomenon was further confirmed in our data. Therefore, to quantify differences in anticipatory *T*_*COM*_ at lift onset across experimental conditions, we excluded the first three trials in each condition block from analysis.

### Data processing

Kinematic and kinetic data were resampled offline to 250 Hz, temporally aligned and processed for analysis by custom software written in MATLAB (MathWorks Inc., Natick, MA, USA). All data were first low-pass filtered at 30 Hz with second order, zero-lag Butterworth filters. A small number of trials (0.3%) was excluded from analyses due to temporal misalignment between kinematic and kinetic data.

### Data analysis

#### Object kinematics and task epochs

 Object kinematics was computed from the data obtained through the infrared LED markers (Fig. 1a). Object translation was estimated as the displacement of the origin of the object’s fixed frame, whereas the object orientation was extracted as the Z-Y-X Euler angle from the rotation matrix using the MATLAB function *rotm2eul*.

As shown by previous work, the two epochs of object lift onset and static hold engage distinct sensorimotor mechanisms. At object lift onset, anticipatory control mechanisms dominate and are based on sensorimotor memory of previous manipulations, as no feedback of the object mass distribution is available, yet^6,22,17^. In contrast, sensory feedback is acquired throughout the lift which can be used, if needed, to modify the digit force distribution used at object lift onset, thus leading to a steady-state digit force distribution during object hold^12,2^. Therefore, our analyses focused on these two epochs to gain insights into anticipatory and feedback-based coordination of digit forces.

Object lift onset was identified using the maximum object height as the starting point of our algorithm and defined as the time at which the object’s height was higher than 1 mm above the table and the object’s vertical velocity was greater than 5 mm/s. Object hold phase was defined as a one-second window starting 0.5 s after the *Hold* cue. As subjects were asked to minimize object tilt, we quantified performance of dexterous manipulation by measuring peak object tilt at object lift onset and during object hold. For the former epoch, peak object tilt was quantified as the largest angle of the vertical axis of the grip device relative to the vertical occurring within 250 ms from object lift onset. During object hold, peak object tilt was computed by averaging the object’s angle relative to the vertical throughout the one-second object hold window.

To determine the trial after which task performance became stable, the resultant torque (external torque minus compensatory torque, *T*_*RES*_; see *Effects of digit offset on manipulation force and compensatory torque*) was tested by linear mixed-effects models (LMMs) with three-way fixed effects of *CM* x *Digit Offset* × *Trial*. The data and *Trial* factor were incrementally increased starting from trial 10 to 20, followed by 9 to 20, and so forth, until a significant *Trial* effect was found. We found task performance became stable from trial 4 to 20 for all subjects and conditions.

#### Coefficient of friction estimation

The translational and torsional coefficients of friction at each contact were estimated for constructing the friction cone at each contact to approximate the minimum grasp (normal) force required to prevent object slip^38^. Slip onset was identified as the time of the peak change of the resultant vertical force rate closest to the first drop in the rate of resultant vertical force. This approach was preferred to defining slip onset as the time at which the first peak change in the resultant vertical force occurred, as subjects might reflexively re-grip the object after slip onset. The accuracy of this algorithm was verified by also examining object position data. The translational coefficients of friction at the thumb and index fingertip were quantified as the largest value of the ratio between the vertical tangential and normal forces at the time of slip onset estimated at the thumb and index fingertip. We found no significant difference between the translational coefficient of friction measured at the beginning versus end of the experimental session (t_15_ = 0.078, *p* = 0.9389). Therefore, we averaged these values for each subject for the analysis of grasp and manipulation forces (below). The coefficient of translational friction averaged across subjects was 1.56 ± 0.06. The torsional coefficient at contacts was assumed to be 6.59 times the translational coefficient^39^. These coefficients of friction were used for estimating the minimally-required slip prevention grasp force through an optimization process (see *Estimation of biomechanically-obligatory and non-obligatory portions of grasp force* in Sect. "Materials and Methods").

#### Analysis of grasp and manipulation forces

A stable grasp that can resist any force vectors applied on the grasped object (i.e., a grasp is force-closure^40^) requires internal forces that are exerted inside the friction cone. Unlike *T*_*COM*_, the internal forces have no direct effect on the object kinematics and are only limited by the friction cone, the object rigidity, and the amount of force each digit can exert. Therefore, manipulation and internal forces play very different roles in dexterous manipulation: the former is devoted to controlling object position and orientation (collectively defined as ‘object pose’), whereas the latter is devoted to preventing object slip. The procedures to decompose the contact forces at individual digit contacts into manipulation and internal forces are described below.

All forces measured by the two transducers were first spatially rotated and aligned from individual transducer coordinates to the object coordinate frame fixed at the center of the handle, and defined following the recommendations for reporting kinematic data^41^. Each digit force was assumed to be applied at a point denoted as the center of pressure (*CoP*, $$\left[ {\begin{array}{*{20}c} {CoP_{x} } & {CoP_{y} } & {CoP_{z} } \\ \end{array} } \right]^{T}$$; inset, Fig. 1a) of the force vector application on each graspable surface and computed from the measured forces ($$\mathop{F}\limits^{\rightharpoonup} = \left[ {\begin{array}{*{20}c} {f_{x} } & {f_{y} } & {f_{z} } \\ \end{array} } \right]^{T}$$) and moment of force ($$\mathop{M}\limits^{\rightharpoonup} = \left[ {\begin{array}{*{20}c} {m_{x} } & {m_{y} } & {m_{z} } \\ \end{array} } \right]^{T}$$) using the cross product equality $\mathop{r}\limits^{\rightharpoonup} \times \mathop{F }\limits^{\rightharpoonup} = \mathop{M}\limits^{\rightharpoonup}$ where $$\mathop{r}\limits^{\rightharpoonup} = \left[ {\begin{array}{*{20}c} {CoP_{x} } & {CoP_{y} } & {CoP_{z} } \\ \end{array} } \right]^{T}$$, superscript *T* denotes transpose, and |*CoP*_*z*_| indicates the distance from the graspable surface to the geometrical center of the sensorized vertical handle, i.e., half of the grasp width normal to the grasp surface. The vertical distance between the tip of the thumb and index finger was defined as $\Delta CoP_{y} = CoP_{y}^{IN} {-} CoP_{y}^{TH}$. We note that *∆CoP*_*y*_ is equivalent to the digit placement offset that we either systematically changed across experimental conditions or was chosen by the subject (U condition; Fig. 1c).

The soft-finger contact model assumes that the finger can exert forces in three directions and one moment of force normal to the contact surface^42^. The contact forces and moments of forces exerted by each digit at the contact surface in the digit-contact coordinate frame can be computed from $$\mathop{F}\limits^{\rightharpoonup}$$ and $$\mathop{M}\limits^{\rightharpoonup}$$ in the object coordinate frame with $$\mathop{r}\limits^{\rightharpoonup}$$. The generalized force vector in ℝ^4^: $$\overset{\lower0.5em\hbox{$\smash{\scriptscriptstyle\rightharpoonup}$}} {F} _{C}^{i} = \left[ {f_{x} \quad f_{y} \quad f_{z} \quad m_{z} } \right]^{T}$$ represents the contact force vectors applied on the contact surface at $$CoP^{i} = \left[ {\begin{array}{*{20}c} {CoP_{x}^{i} } & {CoP_{y}^{i} } \\ \end{array} } \right]^{T}$$, where *i* = TH, thumb or IN, index finger, in the digit-contact coordinate frame.

The effect of $$\overset{\lower0.5em\hbox{$\smash{\scriptscriptstyle\rightharpoonup}$}} {F} _{C}^{i}$$ on the object can be determined by transforming them to the object coordinate frame using the adjoint transformation matrix and the wrench basis^20,42,43^. The adjoint transformation matrix was

1 $$Ad_{{g_{i}^{ - 1} }}^{T} = \left[ {\begin{array}{*{20}l} {R^{i} } \hfill & {\quad 0} \hfill \\ {\hat{p}^{i} R^{i} } \hfill & {\quad R^{i} } \hfill \\ \end{array} } \right]$$

where, for each digit, *R*^*i*^ is the rotation matrix from the object frame to the contact frame and $\hat{p}$ is the skew-symmetric matrix of $\mathop{r}\limits^{\rightharpoonup}$. The wrench basis was

2 $$B^{i} = \left[ {\begin{array}{*{20}c} 1 & {\quad 0} & {\quad 0} & {\quad 0} \\ 0 & {\quad 1} & {\quad 0} & {\quad 0} \\ 0 & {\quad 0} & {\quad 1} & {\quad 0} \\ 0 & {\quad 0} & {\quad 0} & {\quad 0} \\ 0 & {\quad 0} & {\quad 0} & {\quad 0} \\ 0 & {\quad 0} & {\quad 0} & {\quad 1} \\ \end{array} } \right]$$

where each column represents elements of $$\overset{\lower0.5em\hbox{$\smash{\scriptscriptstyle\rightharpoonup}$}} {F} _{C}$$ and each row is the element of the generalized force vector acting on the object. Based on the force application locations of the thumb and index fingertip with the soft-finger contact model assumption^20^, the grasp map (G) can be written as:

3 $$G = Ad_{{g_{i}^{ - 1} }}^{T} B^{i} = \left[ {\begin{array}{*{20}c} 1 & {\quad 0} & {\quad 0} & {\quad 0} & {\quad - 1} & {\quad 0} & {\quad 0} & {\quad 0} \\ 0 & {\quad 1} & {\quad 0} & {\quad 0} & {\quad 0} & {\quad 1} & {\quad 0} & {\quad 0} \\ 0 & {\quad 0} & {\quad 1} & {\quad 0} & {\quad 0} & {\quad 0} & {\quad - 1} & {\quad 0} \\ 0 & {\quad - CoP_{z}^{TH} } & {\quad CoP_{y}^{TH} } & {\quad 0} & {\quad 0} & {\quad - CoP_{z}^{IN} } & {\quad - CoP_{y}^{IN} } & {\quad 0} \\ {CoP_{z}^{TH} } & {\quad 0} & {\quad - CoP_{x}^{TH} } & {\quad 0} & {\quad - CoP_{z}^{IN} } & {\quad 0} & {\quad CoP_{x}^{IN} } & {\quad 0} \\ { - CoP_{y}^{TH} } & {\quad CoP_{x}^{TH} } & {\quad 0} & {\quad 1} & {\quad CoP_{y}^{IN} } & {\quad CoP_{x}^{IN} } & {\quad 0} & {\quad - 1} \\ \end{array} } \right]$$

where $CoP_{i}^{TH}$ and $CoP_{i}^{IN}$ denote the points of thumb and index force application, respectively.

The grasp map was used to transform $$\overset{\lower0.5em\hbox{$\smash{\scriptscriptstyle\rightharpoonup}$}} {F} ^{i} _{C}$$ at individual digit contacts to the object coordinate frame and consequently mapped the digit forces to the total resultant forces ($$\overset{\lower0.5em\hbox{$\smash{\scriptscriptstyle\rightharpoonup}$}} {F} _{O}$$) acting on the object, $$\overset{\lower0.5em\hbox{$\smash{\scriptscriptstyle\rightharpoonup}$}} {F} _{O} ~ = ~G\left[ {\overset{\lower0.5em\hbox{$\smash{\scriptscriptstyle\rightharpoonup}$}} {F} _{C}^{{TH}} \quad \overset{\lower0.5em\hbox{$\smash{\scriptscriptstyle\rightharpoonup}$}} {F} _{C}^{{IN}} } \right]^{T}$$. The null space of G was denoted as G_0_. Projecting $$\overset{\lower0.5em\hbox{$\smash{\scriptscriptstyle\rightharpoonup}$}} {F} _{C}^{i}$$ on to G_0_ resulted in the grasp force (*F*_*G*_) defined as $$\overset{\lower0.5em\hbox{$\smash{\scriptscriptstyle\rightharpoonup}$}} {F} _{G} ~ = ~G_{0} G_{0}^{T} \left[ {\overset{\lower0.5em\hbox{$\smash{\scriptscriptstyle\rightharpoonup}$}} {F} _{C}^{{TH}} \quad \overset{\lower0.5em\hbox{$\smash{\scriptscriptstyle\rightharpoonup}$}} {F} _{C}^{{IN}} } \right]^{T}$$, i.e., the internal force that lies in G_0_. As *F*_*G*_ lies within G_0_, the internal torque (T_INTERNAL_) acting on the object due to *F*_*G*_ is always zero. The manipulation force (*F*_*M*_) can, therefore, be computed as $$\overset{\lower0.5em\hbox{$\smash{\scriptscriptstyle\rightharpoonup}$}} {F} _{C}^{i}$$ minus the grasp force, $$\overset{\lower0.5em\hbox{$\smash{\scriptscriptstyle\rightharpoonup}$}} {F} _{M} ~ = \left[ {\overset{\lower0.5em\hbox{$\smash{\scriptscriptstyle\rightharpoonup}$}} {F} _{C}^{{TH}} \quad \overset{\lower0.5em\hbox{$\smash{\scriptscriptstyle\rightharpoonup}$}} {F} _{C}^{{IN}} } \right]^{T} - \overset{\lower0.5em\hbox{$\smash{\scriptscriptstyle\rightharpoonup}$}} {F} _{G}$$. As the grasp tasks were successfully performed in all trials, we assumed all grasps satisfied the force closure criterion^40^. Based on the force closure assumption, in order to maintain proper *F*_*M*_ without slipping, there must exist an *F*_*G*_ to guarantee that contact forces satisfy friction cone constraints. The magnitude of all thumb and index finger force vectors was quantified as $$\left| F \right| = \mathop \sum \limits_{{i = TH,IN}} \left| {\overset{\lower0.5em\hbox{$\smash{\scriptscriptstyle\rightharpoonup}$}} {F} _{C}^{i} } \right|$$. The magnitude of *F*_*G*_ and *F*_*M*_ was computed as $$\left| {F_{G} } \right|~ = ~\mathop \sum \limits_{{~i = ~TH,~IN}} \left| {\overset{\lower0.5em\hbox{$\smash{\scriptscriptstyle\rightharpoonup}$}} {F} _{G}^{i} } \right|$$ and $$|F_{M} | = \mathop \sum \limits_{{i = TH,IN}} \left| {\overset{\lower0.5em\hbox{$\smash{\scriptscriptstyle\rightharpoonup}$}} {F} _{M}^{i} } \right|$$, respectively. The magnitude of a generalized force vector for each digit was calculated as $$\left| {\vec{F}_{general} } \right| = \sqrt {f _{x}^{2} + f _{y}^{2} + f _{z}^{2} }$$. The data clusters of |*F*_*G*_|, |*F*_*M*_| and the minimum *F*_*G*_ required to prevent object slip (see below) are plotted for each experimental condition as an ellipse measured at object lift onset and hold (Fig. 3b). The ellipses are based on clusters of data from trials 4–20 from all subjects in each digit offset condition. The length of the principal axes of each ellipse was computed using principal component analysis. Half-length of the two principal axes denote the standard deviation along the corresponding axes.

#### Effects of digit offset on manipulation force and compensatory torque

Manipulation force (*F*_*M*_) is responsible for both object position (lift) and orientation control. We quantified the contribution of *F*_*M*_ to object position and orientation control by analyzing the modulation of the normal and tangential components of each digit (^*z*^*F*_*M*_ and ^*y*^*F*_*M*_, respectively). We denote the difference between thumb and index finger *F*_*M*_ components as *∆*^*y*^*F*_*M*_ and *∆*^*z*^*F*_*M*_. Equation (4) describes the contribution of *F*_*M*_ to changing object position, whereas Eq. (5) describes the equality of thumb and index finger ^*z*^*F*_*M*_ to prevent the object’s lateral movement:

4 $$^{y} F_{M}^{IN} +^{y} F_{M}^{TH} = W_{object}$$

5 $$^{z} F_{M}^{IN} +^{z} F_{M}^{TH} = 0$$

where the subscript *IN* and *TH* denote index finger and thumb, respectively, and *W*_*object*_ is the object weight. Equation (6) describes the contribution of *F*_*M*_ to object orientation control expressed as the compensatory torque (*T*_*COM*_) required to counter the external torque (*T*_*EXT*_) caused by the added mass (L or R CM) as the resultant moment of force about the *x*-axis:

6 $$T_{COM} = - CoP_{Z} \cdot \Delta^{y} F_{M} + 0.5\Delta CoP_{y} \cdot \Delta^{z} F_{M}$$

The moment arm for the net digit tangential force (*CoP*_*Z*_) is half object width, whereas the moment arm of digit normal forces corresponds to the digit offset (*∆CoP*_*y*_). The resultant torque (*T*_*RES*_) was computed as the sum of *T*_*COM*_ and *T*_*EXT*_. As the task requirement was to attain a vertical pose from object lift onset to static hold, *T*_*RES*_ should be zero to attain the rotational equilibrium about the *x*-axis. Note that, for a given *∆CoP*_*y*_ (digit offset), the CNS has one degree of freedom to produce *T*_*COM*_ through arbitrary combinations of *∆*^*y*^*F*_*M*_ and *∆*^*z*^*F*_*M*_. Therefore, the object maintains a vertical orientation during the lift as long one of many covariation patterns of the two *F*_*M*_ components satisfies the task requirement of generating *T*_*COM*_.

Our previous work^17^ on unconstrained grasping where subjects spontaneously chose relatively small digit offsets on a trial-to-trial basis has shown that successful performance of our manipulation task is attained by distributing these *F*_*M*_ components near the task manifold (see Fig. 8 of Fu et al.^17^). In the present work, changes in digit offset lead to changes in the re-distribution of the *F*_*M*_ components as *T*_*COM*_ remains constant but one of the two moment arms changes (*∆CoP*_*y*_, Eq. (6)).

#### Effects of digit offset on grasp force

We note that the larger the digit offset, the closer the direction of *F*_*G*_ vector (± 43º relative to *z*_O_ axis for the largest digit offsets, L) to the boundary of object slip (± 57º relative to *z*_O_ axis; the edge of friction cone computed from the translational friction coefficient averaged across subjects; Supplementary Fig. S3a) due to the mechanically-obligatory requirement of *F*_*G*_ being in the null space of the grasp map G (see *Analysis of grasp and manipulation forces*). *F*_*G*_ is responsible for preventing object slip by steering the digit force vectors at individual contacts to remain within the friction cone. Therefore, the closer the *F*_*G*_ vector is to the edge of the friction cone, the greater the risk of object slip. In contrast, the re-distribution of *F*_*M*_ components due to the change in digit offset might reduces the risk of object slip through a chain response, which starts with larger digit offsets increasing the moment arm for both ^*z*^*F*_*G*_ and ^*z*^*F*_*M*_. For a given desired *T*_*COM*_, the greater contribution of ^*z*^*F*_*M*_ leads to a decrease ^*y*^*F*_*M*_. In turn, reducing ^*y*^*F*_*M*_ resulted in less risk of object slip, hence a reduction in grasp force to prevent object slip.

#### Estimation of biomechanically-obligatory and non-obligatory portions of grasp force

It is well known that humans can efficiently attain a small safety margin, defines as each digit’s normal force above the minimum required to prevent object slip^1,12,16,44^. To separate biomechanically obligatory from non-obligatory portions of *F*_*G*_, we estimated the minimally-required slip prevention grasp force, *F*_*G*_^*min*^ using an optimization process based on the manipulation force, the grasp map, and the coefficients of friction^45^. The optimization objective was minimization of the magnitude of *F*_*G*_ with translational and torsional friction cone constraints. The magnitude of *F*_*G*_^*min*^ was computed as $$|F_{G}^{{\min }} | = \mathop \sum \limits_{{i= TH,IN}} \left| {\overset{\lower0.5em\hbox{$\smash{\scriptscriptstyle\rightharpoonup}$}} {F} _{G}^{{\min ,i}} } \right|$$. We then defined the excessive grasp force, *F*_*G*_^*EX*^, as the difference between the magnitude of *F*_*G*_ and *F*_*G*_^*min*^, i.e., *F*_*G*_^*EX*^ =|*F*_*G*_|− *F*_*G*_^*min*^.

#### Computation of relative grasp safety margin

We defined relative grasp safety margin (*SM*_*G*_) as the ratio between *F*_*G*_^*EX*^ and *F*_*G*_ to quantify the proportion of excessive force normalized to exerted grasp force. Note that this definition of *SM*_*G*_ is different from how relative safety margin (*SM*) has been defined in the grasping literature^3,46^. Specifically, the traditional definition is based on decomposing the digit force vector into normal and tangential components. As such, *SM*_*G*_ denotes how far the digit force vector is from the boundary of its friction cone at the digit contact. We note that previous grasping literature focused primarily on manipulation of symmetrical objects at zero-digit offsets, where there are no external torque or torques induced by digit forces acting at non-zero digit offsets. Therefore, the magnitude of normal forces above the minimum normal force required to prevent object slip can take any value up to physiological limits with no risk of changing object orientation. In contrast, our *SM*_*G*_ definition uses the *F*_*G*_ component of the digit force vector obtained from the *F*_*M*_* − F*_*G*_ decomposition. Therefore, while *SM* and *SM*_*G*_ both quantify digit force control efficiency, *SM*_*G*_ captures the excessive force above that required to satisfy both object slip prevention and tilt minimization.

### Statistical analysis

All statistical analyses were conducted in RStudio (version 1.3.1093, RStudio Team, 2020) with R (version 4.0.3, R Core Team, 2020). To determine the effects of digit spacing on digit force coordination, linear mixed-effects models (LMMs) were fitted for all dependent variables with the restricted maximum likelihood criterion using the ‘lme4’ package^47^. All models were built with random intercepts and slopes for the random-effect factor *Subject*.

Task performance, defined as *T*_*RES*_*,* was tested by models with three-way fixed effects of *CM* (3 levels: C, R, and L) × *Digit Offset* (continuous variable) × *Trial* (trial 4 through 20). All models investigating the effects of digit offset on digit force modulation were built with two-way fixed-effects of *CM* (2 levels: R and L) ×|*Digit Offset*| (continuous variable). The relation between *∆*^*y*^*F*_*M*_ and *∆*^*z*^*F*_*M*_ was examined by testing *∆*^*y*^*F*_*M*_ using a LMM with two-way fixed effects of *CM* (2 levels: R and L) ×|*∆*^*z*^*F*_*M*_| (continuous variable). To examine the decreasing rate of *F*_*G*_^*min*^, *F*_*G*_, and *F*_*G*_^*EX*^ components with increasing digit offset, the ratio of *F*_*G*_^*min*^ over *F*_*G*_ and the ratio of *F*_*G*_^*min*^ over *F*_*G*_^*EX*^ components were also tested using LMMs with two-way fixed-effects of *CM* (2 levels: R and L) ×|*Digit Offset*| (continuous variable). LMMs with one-way fixed effect of *Time* (2 levels: object lift onset, hold) were used to determine the effect of task epoch on magnitude and coefficient of variation for *F*_*M*_ and *F*_*G*_^*EX*^. The degrees of freedom for the t-tests and corresponding *p*-values were approximated for all LMMs with the Satterthwaite’s method^48^. All significant levels were set at α = 0.01. Bonferroni adjusted post-hoc LMMs were performed when there were interactions.

## Supplementary Information

Supplementary Information.

## Data Availability

All data needed to evaluate the conclusions in the paper are present in the paper and/or the Supplementary Materials. Raw data are available on Open Science Framework (https://doi.org/10.17605/OSF.IO/U7RQS) with no restrictions.
