# Supplementary material for: Distinct sensorimotor mechanisms underlie the control of grasp and manipulation forces for dexterous manipulation
Source: Sci Rep. 2023 Jul 25;13:12037. doi: 10.1038/s41598-023-38870-8 (PMC10368702; doi:10.1038/s41598-023-38870-8)
Supplement: Supplementary file 1 — Supplementary Information. [file 41598_2023_38870_MOESM1_ESM.pdf]

Supplementary Materials for

Distinct sensorimotor mechanisms underlie the control of grasp and manipulation forces for dexterous manipulation

Yen-Hsun Wu,* Marco Santello

School of Biological and Health Systems Engineering,
Arizona State University, Tempe, AZ 85287 USA

*Corresponding author
Email: yenhsunwu@asu.edu

Supplementary Materials

S1. Manipulation task performance: Compensatory torque and peak object tilt.

We used digit forces, torques and centers of pressure to compute the torque subjects exert at object lift onset (compensatory torque, T_{COM})[17]. Optimal minimization of peak object tilt (measured within 250 ms from object lift onset) occurs when T_{COM} is equal to the magnitude of the external torque caused by the object's asymmetrical mass distribution (T_{EXT}), bringing the resultant torque (T_{RES}) to zero. Therefore, T_{RES} at object lift onset is highly and positively correlated with the peak object tilt (**Supplementary Fig. S1a**). Therefore, we used T_{COM} and peak object tilt as our primary and secondary measures, respectively, of learning of our dexterous manipulation task. Subjects learned T_{COM} within the first three trials (**Supplementary Fig. S1b**; for details see *Results* section). Consequently, the average (±S.E.) peak object tilt significantly decreased from 13.18° (± 1.99) on the first trial to 3.92° (±0.69) from trial 4 onwards.

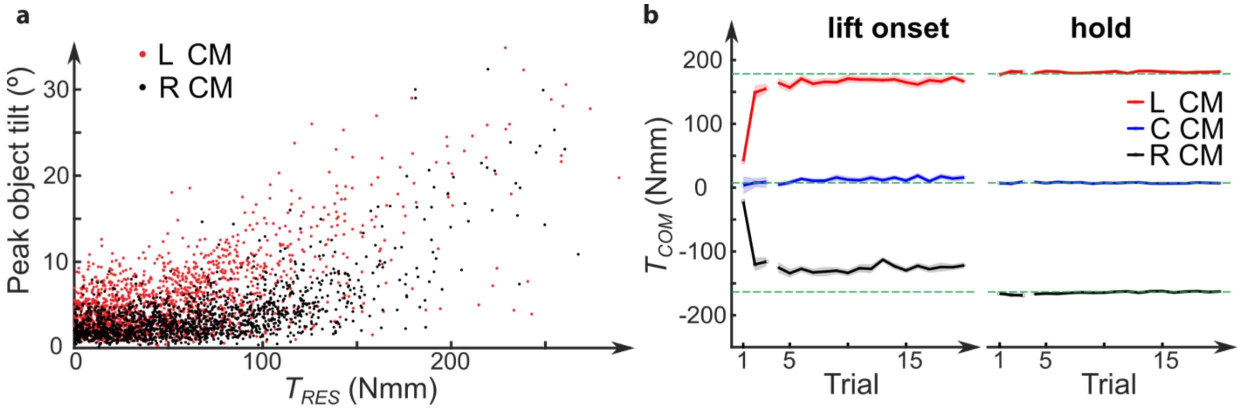

Supplementary Figure S1. *Performance of dexterous manipulation task.* **a** Peak object tilt is plotted against the residual torque (T_{RES}) at object lift onset. Data from left and right center of mass conditions (L and R CM, respectively) are from all trials, subjects and experimental conditions. **b** Compensatory torque at object lift onset and during hold from each trial and center of mass condition averaged across digit offsets and subjects. Shaded areas denote standard error of the mean. Dashed green lines denote the external torque caused by the added mass.

S2. Relative grasp safety margin.

The effects of digit offset on the relative grasp safety margin (SM_G) at object lift onset and during hold is shown in **Supplementary Figure S2a** and **b**, respectively.

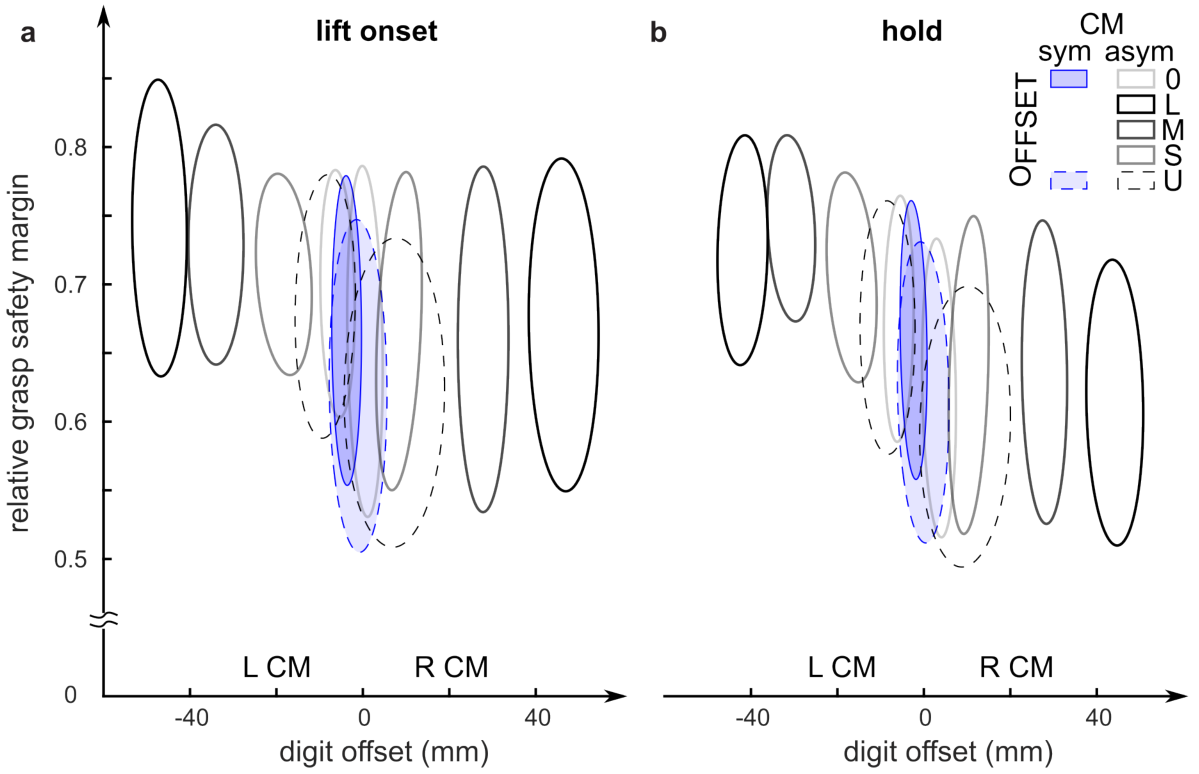

Supplementary Figure S2. *Relative grasp safety margin.* Data in **a** and **b** are relative grasp safety margins from all experimental conditions measured at object lift onset and during hold, respectively. Data (trials 4-20, all subjects) are shown in the same format as **Figure 3**.

S3. Coordination between F_M and F_G.

Subjects tended to use increasingly large F_G relative to F_G^{min} with increasing digit offset. To understand the factors that might have led to this phenomenon, we examined the relation between normal and tangential components of F_G^{EX} (${}^zF_G^{EX}$ and ${}^yF_G^{EX}$, respectively) as a function of digit offset. Increasing digit offset must be accompanied by a parallel increase and decrease in ${}^zF_G^{EX}$ and ${}^yF_G^{EX}$, respectively (red and blue dotted lines, **Supplementary Fig. S3a**) to changes in the direction of the F_G vector. Analysis of the ratios between F_G^{min} and F_G^{EX} components revealed that subjects modulated both components at the same rate as F_G^{min} (at lift onset: $t_{15.17} = -0.14$, $p = 0.893$; hold: $t_{14.59} = -1.89$, $p = 0.079$). Therefore, even though ${}^zF_G^{EX}$ decreases as the same rate as F_G^{min}, the parallel increase of ${}^yF_G^{EX}$ resulted in a slower rate of decrease of F_G^{EX} than the rate at which F_G^{min} decreased with increasing digit offset. We note that, if subjects intended to reduce F_G^{EX} at the same rate as F_G^{min}, they could have reduced each F_G^{EX} component at a higher rate than F_G^{min} (see examples of efficient versus inefficient F_G^{EX} modulation to changes in F_G^{min}, **Supplementary Fig. S3b**).

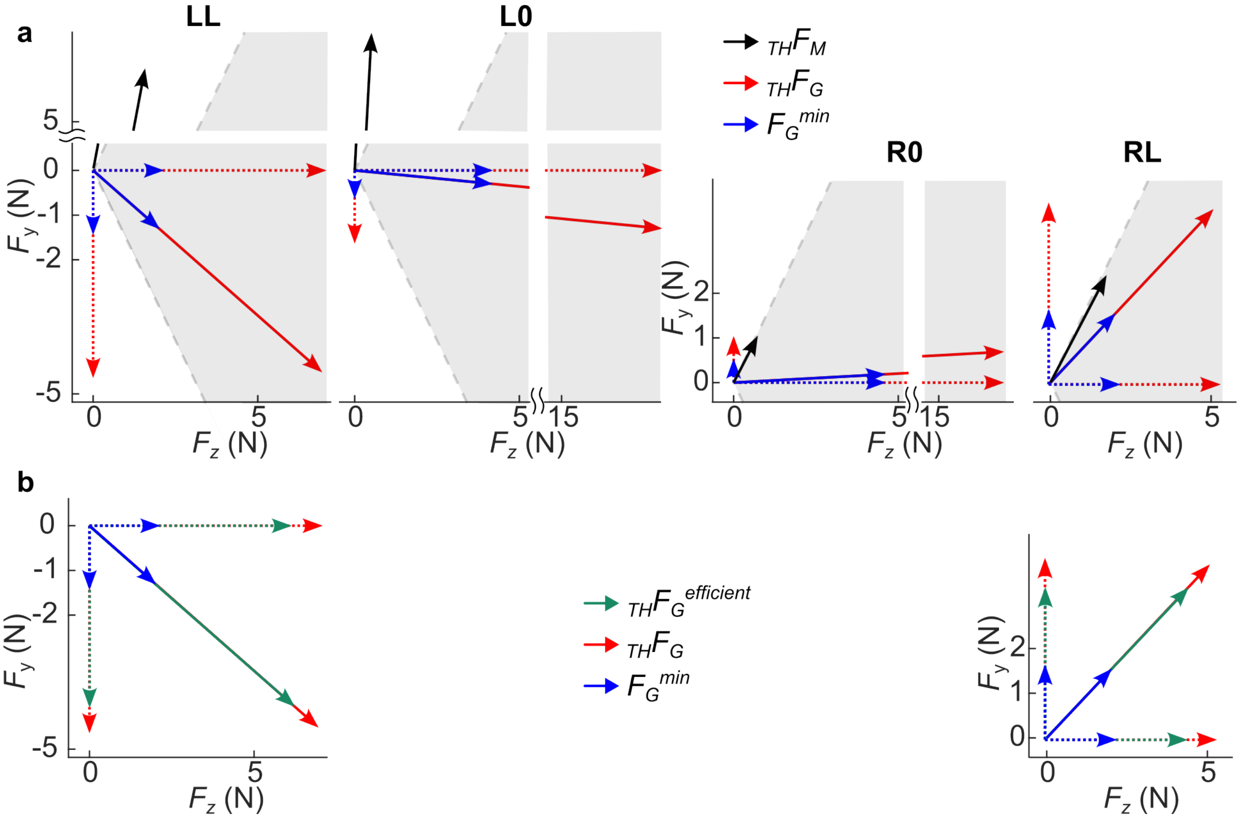

Supplementary Figure S3. *Coordination between F_M and F_G.* **a** Thumb F_M, F_G, and F_G^{min} vectors are plotted in the *y-z* plane as solid black, red, and blue arrows, respectively. Dotted red and blue arrows denote tangential and normal components of F_G and F_G^{min}, respectively. Grey areas denote the friction cone. Data are from one representative trial (trial 4) performed by a representative subject (S10) for the zero and large digit offset condition and both CM conditions (L0 and LL; R0 and RLs). **b** Hypothetical efficient control of F_G ($F_G^{efifcient}$) such that, with increasing digit offset, F_G decreases at the same rate as F_G^{min}. The hypothetical $F_G^{efifcient}$ vector and its components are plotted in green arrows (solid and dashed, respectively) together with experimental data shown in **a** for the LL and RL conditions (left and right plot, respectively).
